# Projecting the impact of variable MDR-TB transmission efficiency on long-term epidemic trends in South Africa and Vietnam

**DOI:** 10.1038/s41598-019-54561-9

**Published:** 2019-12-02

**Authors:** Phillip P. Salvatore, Emily A. Kendall, Dena Seabrook, Jessie Brown, George H. Durham, David W. Dowdy

**Affiliations:** 1 Department of Epidemiology, Johns Hopkins Bloomberg School of Public Health, 615 North Wolfe Street, Baltimore, MD 21205 USA; 2 Division of Infectious Diseases, Johns Hopkins School of Medicine, 733 North Broadway, Baltimore, MD 21205 USA; 3 Linksbridge SPC – 808 Fifth Avenue North, Seattle, Washington 98109 USA; 4 Department of International Health, Johns Hopkins Bloomberg School of Public Health, 615 North Wolfe Street, Baltimore, MD 21205 USA

**Keywords:** Policy and public health in microbiology, Tuberculosis, Antimicrobial resistance

## Abstract

Whether multidrug-resistant tuberculosis (MDR-TB) is less transmissible than drug-susceptible (DS-)TB on a population level is uncertain. Even in the absence of a genetic fitness cost, the transmission potential of individuals with MDR-TB may vary by infectiousness, frequency of contact, or duration of disease. We used a compartmental model to project the progression of MDR-TB epidemics in South Africa and Vietnam under alternative assumptions about the relative transmission efficiency of MDR-TB. Specifically, we considered three scenarios: consistently lower transmission efficiency for MDR-TB than for DS-TB; equal transmission efficiency; and an initial deficit in the transmission efficiency of MDR-TB that closes over time. We calibrated these scenarios with data from drug resistance surveys and projected epidemic trends to 2040. The incidence of MDR-TB was projected to expand in most scenarios, but the degree of expansion depended greatly on the future transmission efficiency of MDR-TB. For example, by 2040, we projected absolute MDR-TB incidence to account for 5% (IQR: 4–9%) of incident TB in South Africa and 14% (IQR: 9–26%) in Vietnam assuming consistently lower MDR-TB transmission efficiency, versus 15% (IQR: 8–27%)and 41% (IQR: 23–62%), respectively, assuming shrinking transmission efficiency deficits. Given future uncertainty, specific responses to halt MDR-TB transmission should be prioritized.

## Introduction

The global epidemic of multidrug resistant tuberculosis (MDR-TB) represents a major challenge to worldwide TB control efforts. In 2016, an estimated 490,000 cases of MDR-TB occurred, representing 4% of new TB cases and 19% of previously-treated TB cases globally^1^. While substantial efforts have been made to improve the detection and effective treatment of patients suffering from drug resistant TB, the global burden of MDR-TB has not abated^1^. As MDR-TB epidemics may continue despite improvements in the diagnosis and treatment of drug susceptible TB (DS-TB), successful TB control may increasingly depend on the trajectory of MDR-TB. If MDR-TB fails to transmit efficiently, improved DS-TB management alone could be sufficient to contain epidemics of MDR-TB, but if MDR-TB transmits nearly or equally as efficiently, it may replace DS-TB as the dominant TB strain^2^. However, predicting these epidemic trajectories remains a challenge.

The relative transmission efficiency of MDR-TB has classically been conceptualized as a genetic “fitness cost,” a reduction in reproductive capacity that may accompany the development of drug resistance. Recent laboratory evidence, however, suggests that not all resistance-conferring mutations carry a fitness cost and that such costs may be offset by compensatory mutations, leading to high-fitness MDR strains^3^. Epidemiological time-series studies indicate that the prevalence of MDR-TB isolates carrying these low-cost or compensatory mutations has increased over time in several settings^4,5^. Simultaneously, evidence has accumulated that modern MDR-TB epidemics are driven primarily by transmission rather than by the acquisition of drug resistance during treatment^2,6,7^. Nevertheless, it is possible that the epidemiological transmission potential of MDR-TB (as might be estimated, for example, in calculations of the basic reproductive number R_0_) may differ from that of DS-TB^8^. For example, individuals with MDR-TB may have lower infectiousness (e.g., less pulmonary concentration of bacilli) or fewer susceptible contacts (e.g., they are more socially isolated and thus make fewer infectious contacts)^9,10^ than individuals with DS-TB. We therefore utilize the term “relative transmission efficiency,” describing the relative frequency of generating secondary infections per unit of infectious person-time (comparing MDR-TB to DS-TB), to capture the biological as well as social and epidemiological factors which influence MDR-TB transmission. The future trajectory of MDR-TB (and the relative importance of efforts to control MDR-TB) is likely to depend substantially on the relative efficiency with which MDR-TB cases transmit infection.

In light of the complexity of empirical evidence^3,11^ regarding MDR-TB transmission efficiency and the variety of modeling approaches^7,12,13^ used to evaluate this phenomenon, we sought to compare how different assumptions about MDR-TB transmission efficiency influence projections of long-term MDR-TB incidence. We used a mathematical model of DS-TB and MDR-TB to project future trajectories of MDR-TB epidemics in South Africa and Vietnam under three competing assumptions about the relative transmission efficiency of MDR-TB. Within this framework, we first explore the relative ability of models using these different assumptions to recapitulate empirical DS-TB and MDR-TB incidence data. We then show that models using these assumptions forecast highly divergent epidemic trajectories of MDR-TB.

## Methods

### TB dynamics and natural history

**Figure 1:**
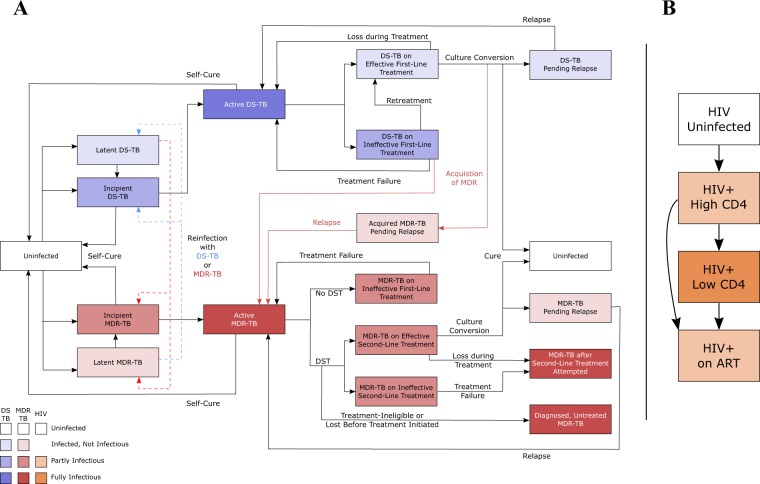
Model Structure. States of TB infection and possible transitions between them are represented in panel A. Compartment colors correspond to the respective infectiousness and TB-associated mortality of each state. In addition to TB natural history, populations are also classified by treatment history (treatment-naïve or previously-treated, not shown), and HIV status (represented in panel B). Following HIV infection, populations transition through states of increasing immunosuppression or to antiretroviral therapy (ART) at defined rates. Populations returning to an uninfected state through self-cure remain classified as treatment-naïve; other distinctions between uninfected compartments are shown only for illustrative purposes.

To explore the impact of differential transmission efficiency on epidemic projections, we formulated a deterministic compartmental model of adult TB transmission^14^. We then used this model to independently simulate epidemics of TB in two high-burden countries. Our model represents the natural history of DS-TB and MDR-TB as follows (Fig. 1A): susceptible populations who become infected with either DS-TB or MDR-TB may develop latent TB infection (and slow progression to active TB disease) or rapidly progress to active TB. Regardless of whether populations progress rapidly or slowly, all experience a period of incipient disease (where cases are not ostensibly symptomatic but are partially infectious) before developing clinically detectable TB. The emergence of MDR-TB occurs initially through natural selection during first-line treatment of DS-TB but subsequently spreads through person-to-person transmission. Our model incorporates a simplified interaction of HIV and TB co-epidemics (Fig. 1B) with the annual incidence of HIV fitted to setting-specific estimates^15^ of adult HIV incidence. Populations living with HIV are classified into three states: high CD4 (T cell levels above 250 cells/mL), low CD4 (levels below 250 cells/mL), and receiving antiretroviral therapy (ART). A subpopulation’s HIV status in turn affects TB dynamics, including susceptibility, mortality, and care-seeking.

### Transmission efficiency of DS-TB and MDR-TB

We investigated the impact of transmission-related differences between DS-TB and MDR-TB by varying assumptions about the relationship between different strains’ transmission efficiency over time. We define transmission efficiency as the mean number of new TB infections that occur during each infectious person-year of active TB. (This quantity includes infections that eventually progress to disease and infections that never progress to disease, as opposed to the reproductive number which quantifies the number of secondary cases.) Transmission efficiency captures both biological processes of TB transmission (infectious doses, bacterial virulence, etc.) and non-biological processes (contact rates, population mixing, etc.). Therefore, the transmission efficiency of either DS- or MDR-TB strain may be increased through changes in underlying biology or through population-level influences. In our approach, we concentrate on the relative transmission efficiency of MDR-TB; that is, the degree to which fewer MDR-TB infections will arise than DS-TB infections under comparable periods of infectious person-time. Mathematically, this is accomplished by setting the transmission parameter for MDR-TB equal to the product of the transmission parameter for DS-TB and a relative efficiency term (domain [0, 1]). (See “Sampling & Calibration” below and “Model Summary” in the *Appendix* for technical and sampling details.)

**Figure 2:**
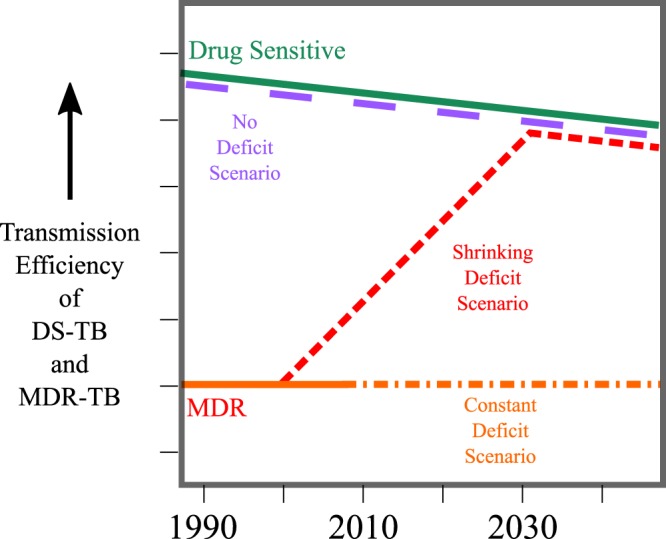
Transmission Efficiency Scenarios. The assumed transmission efficiency (transmission events per 1,000 infectious person-years) of DS-TB over time is drawn in green; the downward slope recapitulates reductions in TB transmission efficiency due to secular trends unrelated to MDR-TB diagnosis and treatment (for example, reductions in crowding, improved socioeconomic conditions, etc.). In our three model scenarios, we assume either that the transmission efficiency of MDR-TB is at a perpetual deficit compared to that of DS-TB (Constant Deficit Scenario, drawn in orange); that the transmission efficiency of MDR-TB is consistently the same as that of DS-TB (No Deficit Scenario, drawn in magenta); or that MDR-TB has lower transmission efficiency than DS-TB initially but gradually converges towards that of DS-TB over time (Shrinking Deficit Scenario, drawn in red). Years are shown for illustrative purposes; dates of MDR-TB emergence and rates of increase/decrease in transmission efficiency are sampled from defined ranges; see Sampling & Calibration for further details.

We evaluated three plausible transmission efficiency scenarios (illustrated in Fig. 2). In the first scenario (“Constant Efficiency Deficit”), we assume that MDR-TB emerges historically with a deficit in transmission efficiency (relative to that of DS-TB) which remains constant through the present and into the future. In the second scenario (“Shrinking Efficiency Deficit”), we assume that MDR-TB emerges with a deficit in transmission efficiency which gradually shrinks (through, for example, compensatory evolution or social processes that concentrate MDR-TB in high-transmission settings) until the efficiency of MDR-TB equals that of DS-TB. In the third scenario (“No Efficiency Deficit”), we assume that MDR-TB emerges historically with a transmission efficiency equal to that of DS-TB and continues to transmit with equal efficiency over time.

The date of emergence of MDR-TB was varied from 1971 to 1996, reflecting uncertainty in the timing of emergence of modern MDR-TB strains (see Table S3). The initial transmission efficiency of MDR-TB relative to that of DS-TB was similarly varied from 38–94% in the Constant Deficit and Shrinking Deficit scenarios. In the Shrinking Deficit scenario, the annual rate of reduction in the MDR-TB transmission efficiency deficit was varied from 0 to 1·5% per year. Further details on the parameterization of each scenario may be found in the *Appendix*.

### Diagnosis & treatment

Our model conceptualizes treatment as being either sufficient to ultimately achieve cure (“effective”) or of a nature that does not cure active TB by the end of therapy (“ineffective”). Upon diagnosis with TB, therapy (either effective or ineffective) may be initiated following a mean diagnostic delay. Patients with DS-TB who receive effective therapy undergo a cessation of infectiousness^16^, a reduction in mortality, and – upon treatment completion – long-term cure or eventual relapse. Those receiving ineffective therapy (e.g., patients with MDR-TB receiving first-line treatment) experience partially reduced infectiousness and TB-associated mortality. Patients with MDR-TB receive drug susceptibility testing (DST) at a level determined by setting-specific national estimates of DST coverage over time (see Fig. S2)^1^. Those who do not receive DST before treatment initiate ineffective first-line therapy, while those who receive DST initiate longer second-line therapy which may be effective or ineffective. Patients with MDR-TB who receive effective second-line therapy experience partially reduced infectiousness and TB-associated mortality for the first six months of treatment (reflecting potential delays in initiating second-line therapy and reduced potency of historical second-line agents), followed by cessation of infectiousness and reduced mortality for the remainder of the treatment regimen. Patients may prematurely stop any treatment regimen; this probability is correlated with the length of each regimen (see Table 1). Rates of ART initiation are calculated from setting- and time-dependent data on ART coverage, and TB/HIV co-infected patients in whom either disease is detected experience increased rates of treatment initiation for the other condition (see the *Appendix* for modeling methods and ordinary differential equations).

**Table 1:** Selected* Parameter Values.

Description	Median	Sampling Range^†^	Distribution	References
Probability of rapid progression after initial tuberculosis infection	0·14	0·08–0·25	Logit-normal	^45^
Reactivation rate from latent to incipient active tuberculosis, per year	0·001	0·0005–0·002	Lognormal	^46–49^
Rate of tuberculosis diagnosis and treatment initiation, per year	1·0	0·7–1·5	Lognormal	^1,50,51^
Proportion failing to initiate treatment for multidrug-resistant tuberculosis after diagnosis (in excess of loss to follow-up of patients with drug-susceptible tuberculosis)	0·05	0·02–0·10	Logit-normal	^1,51^
**Proportion of treated patients who have an apparent treatment response**				
Newly diagnosed patients with drug-susceptible tuberculosis, first-line therapy	0·98	0·96–0·99	Logit-normal	^1,52–54^
Patients with multidrug-resistant tuberculosis, longer therapy	0·77	0·66–0·85	Logit-normal	^55–57^
**Proportion who relapse, among those with apparent treatment response**				
Newly diagnosed patients with drug-susceptible tuberculosis, first-line therapy	0·04	0·026–0·06	Logit-normal	^57,58^
Patients with multidrug-resistant tuberculosis, longer therapy	0·04	0·015–0·10	Logit-normal	^58,59^
**Probability of loss to follow-up during therapy**				
First-line therapy	0·06	0·03–0·10	Logit-normal	^1^
Longer therapy for multidrug-resistant tuberculosis	0·11	0·04–0·25	Logit-normal	^60,61^
Risk of acquiring multidrug resistance during first-line therapy	0·004	0·0015–0·01	Logit-normal	^56^

^*^Parameters were selected for inclusion in this table based on familiarity to a scientific audience and prior belief of strong association with MDR-TB transmission. See the *Appendix* for a full listing of all parameters, sampling distributions, and references. ^†^Sampling ranges represent the 2·5th to 97·5th percentiles of unbounded distributions and lower to upper bounds of uniform distributions.

### Sampling & calibration

For each input parameter used in our model, we defined a range of plausible values based on the scientific literature (Tables 1 and S1–S3). These ranges were parameterized as lognormal (for continuous ranges bounded at 0), logit-normal (for continuous ranges bounded between 0 and 1), or uniform probability distributions (for parameters on continuous ranges with sparse support in empirical literature). To capture the uncertainty in these parameter values, we utilized a two-stage, semi-Bayesian Sampling/Importance Resampling algorithm to simulate epidemics consistent with empirical data^17^.

Using Latin Hypercube Sampling, we generated 135,000 sets of initial parameters, each set composed of one value for each parameter related to DS-TB drawn from the probability density defined in Table 1 (see Tables S1–S3 for all parameters). Each parameter set was then used to simulate a DS-TB epidemic to 1990 followed by a DS-TB/HIV epidemic to 2016. In the first stage of calibration, these simulations were fitted to the WHO estimates of total TB incidence in each setting and setting-specific estimates of the proportion of HIV-positive patients among incident TB cases (using national survey data in South Africa and WHO estimates in Vietnam)^1,18^. Each calibration point was defined as a bounded beta distribution, and a pseudo-likelihood weight for each initial parameter set was defined as the joint probability density of the simulated DS-TB epidemic incidence and proportion of HIV-positive patients. (See Table S4 and Fig. S4 for DS-TB calibration targets and results).

In the second stage of sampling, 135,000 DS-TB/HIV parameter sets were resampled according to their pseudo-likelihood weights, and accompanying values for MDR-TB parameters in each set were drawn from their defined distributions (Table 1), again using Latin Hypercube Sampling. These parameter sets were used to simulate new DS-TB/HIV/MDR-TB epidemics from the date of the emergence of MDR-TB (itself a sampled parameter value) until 2016. In the second stage of calibration, these simulations were fitted to the proportion of MDR-TB among recently-diagnosed TB in new patients and, separately, in previously-treated patients (as measured in national drug resistance surveys)^19–21^. Each calibration point was defined as an independent normal distribution, and a new pseudo-likelihood weight for each parameter set was calculated from the MDR-TB calibration targets (see Table S4). These pseudo-likelihood weights were used to resample those DS-TB/HIV/MDR-TB parameter sets most consistent with historical MDR-TB epidemics.

### Outcomes and statistical analysis

For projections of our primary outcomes, we report median values as well as uncertainty ranges (UR), defined as the 5^th^ and 95^th^ percentiles of posterior distributions. The relative fit of each transmission scenario to the observed epidemiological data after calibration was determined by Bayes Factors (BFs); a BF is calculated as the ratio of the cumulative posterior probabilities of two models and can be interpreted as the calibration data’s support for one model over another. Unless otherwise stated in the text, BFs were calculated in support of the Constant Deficit scenario relative to other models.

## Results

**Figure 3:**
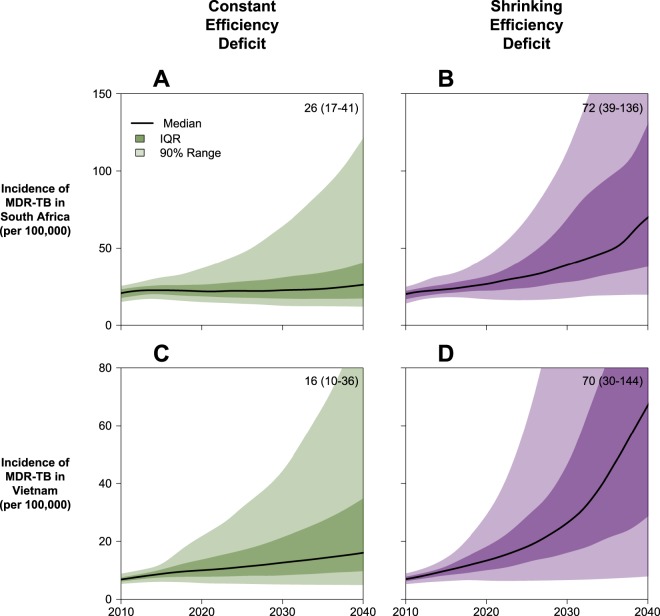
Projections of MDR-TB Incidence in South Africa and Vietnam. Simulated MDR-TB epidemics in South Africa and Vietnam were projected from 2010 to 2040. Panels A and B illustrate the projections of each scenario in South Africa, while panels C and D illustrate the projections of each scenario in Vietnam. The 2040 projected median (IQR) values are included in the upper right of each panel. IQR represents 25th to 75th percentiles and the 90% range represents the 5th to 95th percentiles of posterior simulations.

We projected the absolute and relative incidence of MDR-TB in 2040 in South Africa and Vietnam under three alternative scenarios. We estimated both the absolute and relative incidence of MDR-TB to be substantially lower if the transmission efficiency of MDR-TB remains constant (“Constant Deficit”) than if the transmission efficiency of MDR-TB increases over time (“Shrinking Deficit”). For example, in South Africa, the Constant Deficit scenario (Fig. 3A) predicts the incidence of MDR-TB in 2040 at 26 cases per 100,000 (IQR: 17–41), comprising 5% (IQR: 4–9%) of all incident TB (see also Figs. S5 and S7), a median 2·0-fold increase over 2016. By contrast, projections from the Shrinking Deficit scenario (Fig. 3B) predict a much higher MDR-TB incidence, reaching a median of 72 cases per 100,000 (IQR: 39–136) by 2040 and accounting for 15% of incident TB (IQR: 8–27%), a median 5·1-fold increase. Similar trends were predicted in Vietnam (Fig. 3C,D), where the Constant Deficit scenario predicts an MDR-TB incidence of 16 per 100,000 (IQR: 10–36) in 2040, comprising 14% (IQR: 9–26%) of incident TB, a 2·2-fold increase (IQR: 1·6–3·6-fold) over 2016. By contrast, the Shrinking Deficit scenario predicts an incidence of 70 per 100,000 (IQR: 30–144), accounting for 41% (IQR: 23–62%) of incident TB, a 5·4-fold increase (IQR: 3·6–7·4-fold) over 2016. The No Deficit model was consistently supported least well by the data (see below) but projected median MDR-TB incidences of 130 per 100,000 in South Africa and 24 per 100,000 in Vietnam by 2040 (see Fig. S6).

### Calibration and model fit

**Figure 4:**
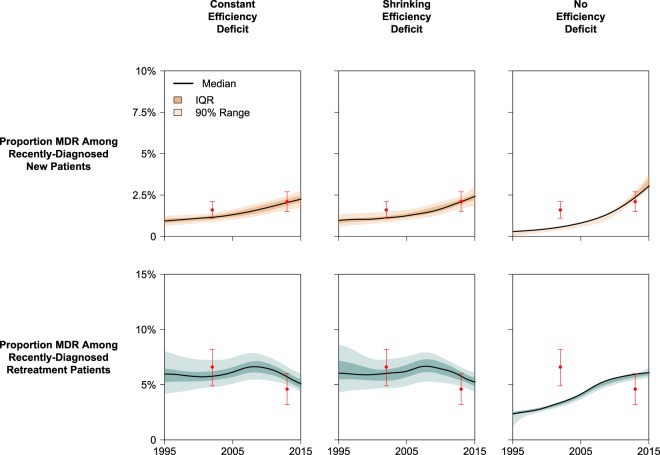
Calibration Performance for South Africa. Simulated epidemics are weighted according to how well each reproduced empirical calibration targets (historical estimates of MDR in new and previously-treated TB cases). Recent diagnoses are defined as any populations transitioning from active, untreated TB into any diagnosis/treatment state. Red points represent median and 95% confidence intervals for calibration targets drawn from national survey data. IQR represents 25th to 75th percentiles and the 90% range represents the 5th to 95th percentiles of posterior simulations.

**Figure 5:**
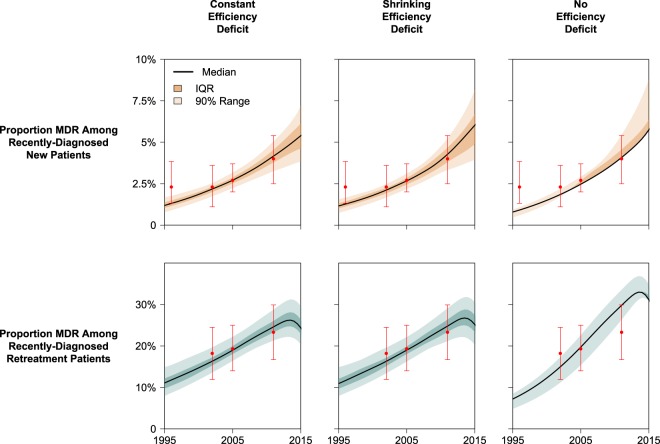
Calibration Performance for Vietnam. Simulated epidemics are weighted according to how well each reproduced empirical calibration targets (historical estimates of MDR in new and previously-treated TB cases). Recent TB diagnoses were used (instead of incident TB cases) to better represent the sampling methodologies used in national drug resistant surveys which were used for calibration. (The 1996 prevalence survey in Vietnam measured the proportion MDR in new cases only.) Recent diagnoses are defined as any populations transitioning from active, untreated TB into any diagnosis/treatment state. Red points represent median and 95% confidence intervals for calibration targets drawn from national survey data. IQR represents 25th to 75th percentiles and the 90% range represents the 5th to 95th percentiles of posterior simulations.

To confirm that these model projections offered accurate reproductions of historical data from South Africa and Vietnam, we used statistical measurements (Bayes Factors) to compare the fit of our model calibration in each scenario. Consistent with empirical observations, our model of South Africa demonstrates increasing TB incidence between 1990 and 2005 (driven largely by trends in HIV coinfection) with MDR-TB composing less than 1·5% of all TB cases by 2016 (Fig. 4 and *Appendix* Fig. S4). In contrast, our model of TB in Vietnam (where HIV coinfection is uncommon) demonstrates a slowly declining TB epidemic with MDR-TB accounting for roughly 20% of previously-treated TB cases by 2016 (Figs. 5 and S4). In both country settings, epidemiological data could be reproduced with similar accuracy in the Constant Deficit and Shrinking Deficit models (BF = 2·6 in South Africa, BF = 2·1 in Vietnam; see Table S5). The No Deficit model was supported far more poorly by the empirical data (BF < 10^−8^ and BF < 10^−3^ relative to other scenarios in South Africa and Vietnam, respectively).

**Figure 6:**
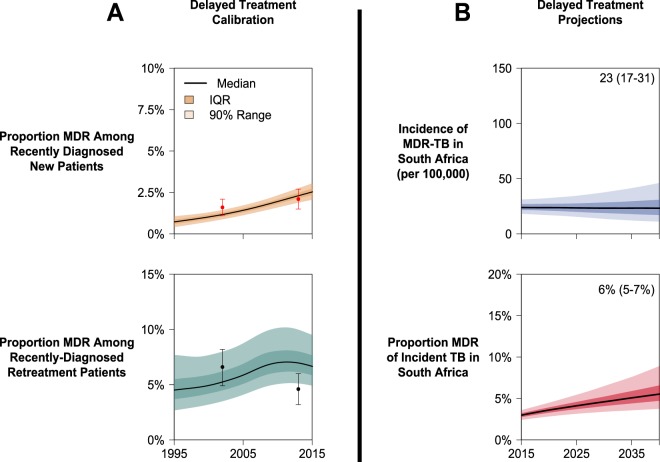
Replication of Previous Findings. The calibration results of an alternative (“Delayed Treatment”) scenario in South Africa – in which we assumed a median delay of 10 years prior to treatment initiation –are represented in panel A. Points represent median and 95% confidence intervals for calibration targets drawn from national survey data. For this scenario only, calibration excluded data on previously-treated TB patients, consistent with methods used in the replicated publication^7^. Simulated MDR-TB epidemics in South Africa under the Delayed Treatment scenario projected to 2040 are represented in panel B. IQR represents 25th to 75th percentiles and the 90% range represents the 5th to 95th percentiles of posterior simulations.

Under the poorly-supported No Deficit scenario, our model projected that MDR-TB in South Africa would comprise 22% (IQR: 11–51%; Fig. S5) of all TB incidence by 2040, much higher than the estimate of 5·7% (95% UR: 3·0–7·6%) in a recent model that used a similar assumption of no transmission efficiency deficit^7^. By increasing the estimated median delay between the onset of TB disease and the initiation of care from 12 months (our initial value) to 10 years (as reported in the prior publication), our model recapitulated the results of the previous model (Fig. 6). Specifically, when assuming a 10-year duration of illness, we projected that MDR-TB will account for 6% (IQR: 5–7%) of incident TB in South Africa by 2040, comparable to the previous estimate of 5·7%^7^, suggesting that the discrepancy in results may not be due to fundamental differences in model structures or statistical approaches but can be explained largely by different assumptions about the duration of illness before treatment initiation. See the *Appendix* for further details.

### Sensitivity analyses

**Figure 7:**
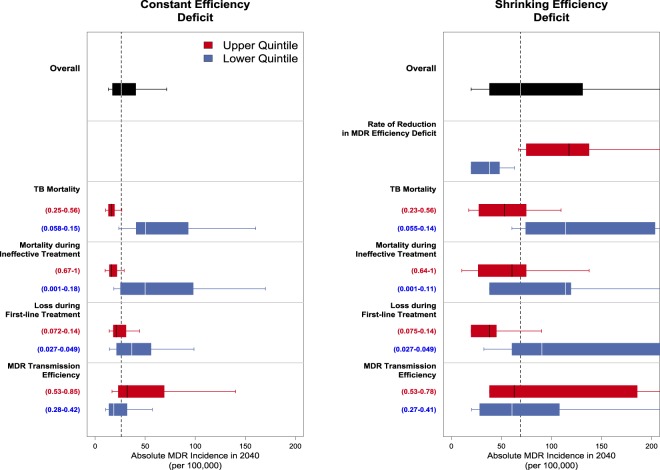
Sensitivity Analysis – Influence of Key Model Parameters on Projections of MDR-TB Incidence in South Africa. The top 5 parameters which most strongly impact the distributions of MDR-TB incidence in 2040 in projections of the epidemic in South Africa are displayed. Each boxplot represents the distribution of values for the primary outcome (the incidence of MDR-TB in 2040) within a given set of simulations. Pairs of boxplots represent groups of simulations categorized by values of a single input parameter: red boxplots represent the outcomes of those simulations with parameter values in the upper 20% of all simulations; blue boxplots represent the outcomes of those simulations with parameter values in the lower 20% of all simulations. More influential parameters demonstrate a greater separation of the distributions of outcome between simulations in the upper quintile and simulations in the lower quintile of parameter values. To the left of each panel are included the input parameter values corresponding to the accompanying quintile. In black is represented the overall distribution of the outcome across all simulations and the median estimate is drawn as a vertical dotted line. Boxes represent the median, 25th, and 75th percentiles of the distribution of outcomes; whiskers represent the 5th and 95th percentiles of the distribution of outcomes. In the Constant Deficit model, parameters involving the reduction in MDR-TB transmission efficiency deficit are excluded by definition.

The variability in MDR-TB epidemic projections was influenced most strongly by parameters determining the transmission and mortality of MDR-TB (see Fig. 7 for South Africa). In the Shrinking Deficit model, the rate of increase in MDR-TB transmission efficiency was highly correlated with projected 2040 MDR-TB incidence (upper vs. lower quintile medians: 117 vs. 38 per 100,000). This parameter is the key dynamic driving the difference between the modest increases in MDR-TB incidence in the Constant Deficit scenario and the sizeable increases in incidence in the Shrinking Deficit scenario. In the Constant Deficit model, the relative efficiency of MDR-TB transmission was similarly correlated with future incidence (upper vs. lower quintile medians, 32 vs. 19 per 100,000). In the Shrinking Deficit and Constant Deficit models, both TB mortality rates and loss during first-line treatment (which determine infectious MDR-TB person-time in the absence of second-line therapy) were strongly correlated with projected MDR-TB incidence. Additional parametric and nonparametric sensitivity analyses may be found in the *Appendix*, as well as parameter value posterior distributions (Figs. S11–S16).

## Discussion

This analysis illustrates that the future trajectories of MDR-TB in South Africa and Vietnam are highly dependent on the transmission efficiency of MDR-TB and its trend over time. Assuming a constant deficit in transmission efficiency, the absolute incidence of MDR-TB was projected to increase only modestly by 2040 – to 26 per 100,000 in South Africa and to 16 per 100,000 in Vietnam. If the transmission efficiency of MDR-TB increases over time, however, the incidence of MDR-TB could rise as high as 72 per 100,00 in South Africa and 70 per 100,000 in Vietnam. These findings underscore the importance of additional research to better estimate the relative transmission efficiency of MDR-TB from an epidemiological (i.e., not purely genetic or *in vitro*) perspective, including evaluation of multiple settings and trends over time.

Much of the scientific literature examining the fitness costs and compensatory evolution of drug resistant *M. tuberculosis* originates from laboratory studies, which have provided strong evidence of the potential for compensatory mutations to overcome biological fitness costs of mutations conferring resistance to isoniazid, rifampin, and ethambutol^22–24^. Unfortunately, laboratory fitness assays are imperfect models of human transmission^25^, and evidence of the potential for changes in the transmission efficiency of MDR-TB remains sparse. In this model of South Africa and Vietnam, we found little support for scenarios in which the epidemiological transmission efficiency of MDR-TB has historically equaled that of DS-TB, though scenarios in which the transmission efficiencies of MDR-TB and DS-TB converge over time were more plausible.

To empirically estimate the relative transmission efficiency of MDR-TB, we must rely primarily on indirect evidence from studies of incident and prevalent TB in human populations^26^. For example, studies of household TB contacts disagree whether more secondary cases arise from index MDR-TB or DS-TB patients^27–29^. Importantly, while the transmission efficiency of MDR-TB may increase due to evolutionary adaptation, it may also increase (or decrease) as a result of changes in contact rates, mixing patterns, and other population-level characteristics– and this transmission efficiency may be different in different settings (as allowed in our model between South Africa and Vietnam). For example, the clustering of MDR-TB in incarcerated populations and hospital settings (leading to increased transmission) is well documented^30–32^, and such clustering may change over time as diagnostic and treatment practices evolve; these changes in clustering may easily be differential across countries or other geographical regions.

Additional studies may further increase our understanding of MDR-TB transmission efficiency in the future. Novel biomarkers and diagnostic assays may help determine the onset of TB infectiousness, evaluate the trajectory of infectiousness over the course of disease^33,34^, and better document newly acquired infection^35,36^. Additional important sources of data will be the continuation and expansion of repeated national drug resistance surveys^19–21^. Without a clear understanding of trends in transmission efficiency, our results indicate that the future of MDR-TB epidemics will remain uncertain. Given the potential for dramatic expansion of MDR-TB epidemics, the inability of classical TB interventions to curtail MDR-TB transmission, and the tremendous economic and human burden imposed by MDR-TB, our results indicate that responses specifically designed to combat MDR-TB epidemics should be prioritized.

Our results offer an important complement to MDR-TB modeling projections published previously. Previous studies have projected that acquired drug resistance accounted for fewer than 10% of MDR-TB cases in 2013, consistent with the results presented here (see Fig. S8)^6^. Importantly, our No Deficit model was poorly supported by empirical data in South Africa and projected greater increases in the burden of MDR-TB than were reported in a previous model of MDR-TB dynamics that incorporated an assumption of no deficit in MDR-TB transmission efficiency^7^. Parameter selection is a delicate task and even minor differences in prior distributions may affect long-term projections^37^. However, in comparing our results to those of a previous model assuming no historical deficit, it is likely that the average duration of TB disease before treatment initiation in South Africa is substantially shorter than 10 years^38–40^, as implicitly assumed in the prior model^7^ and as required by our model to replicate these previous findings. Therefore, similar compartmental models that assume no historical deficit in transmission efficiency may not be realistic representations of the MDR-TB epidemic in South Africa, though it remains plausible that this deficit has closed over time. However, evidence that some MDR-TB epidemics are expanding at a relatively slow pace^1,41^ is less consistent with our Shrinking Deficit model than our Constant Deficit model. These trends may therefore suggest that deficits in MDR-TB transmission efficiency are not shrinking (or shrinking only very slowly) over time.

Our methodology is not without its limitations. South Africa and Vietnam are countries with unique TB epidemics that were chosen based on the availability of repeated national drug resistance survey data. Our future projections had wide uncertainty ranges, reflecting uncertainty in both calibration data and the underlying natural history of MDR-TB. We also calibrated to available data in 2017; subsequent updates to these data have been made and may influence our final results^42^. Our model assumes an exponential age-structured population, which may overestimate the impact of TB treatment^43^ and therefore underestimate future absolute TB incidence; as the same age-structure was use in all three transmission efficiency scenarios, this was unlikely to influence comparisons between Constant and Shrinking Deficit model projections. Sensitivity analyses suggest that our results were largely robust to uncertainty in these parameter values, but our estimates were influenced by parameters determining the infectious person-time of untreated MDR-TB. More precise estimates of these underlying data would reduce uncertainty in long-term projections from future modeling efforts. Additionally, in projecting future epidemics, we assumed that recent trends in the scale-up of MDR-TB diagnosis and second-line treatment will continue. As scale-up has been faster in South Africa than Vietnam, the projected relative incidence of MDR-TB increases more gradually in South Africa in all scenarios (see *Appendix* Fig. S5). If these trends either improve or stagnate over time (for example, with the release of novel all-oral regimens for MDR-TB), our projections of future MDR-TB burden will be inaccurate. Finally, in the absence of unambiguous empirical data describing changes in MDR-TB transmission efficiency, in the Shrinking Deficit model we assumed a linear increase over time (see Fig. 2); this temporal trend could take other shapes, with corresponding effects on our model’s projections.

Global efforts to control TB are likely to hinge on the future trajectory of MDR-TB epidemics. While MDR-TB is often described as carrying a fitness cost associated with drug resistance, this characterization may be inaccurate. Alternative arguments suggest that the transmission efficiency of MDR-TB may equal that of DS-TB already or in the future. We investigated the importance of these assumptions in influencing projections of MDR-TB epidemics, finding strong evidence that MDR-TB has been characterized by lower epidemiological transmission efficiency than DS-TB in the past but no suggestion that this historical difference will necessarily continue into the future. A better understanding of these dynamics in human populations will improve our ability to predict (and possibly prevent) increases in the burden of MDR-TB. In the meantime, specific investment in responses specific to MDR-TB (such as expanded DST coverage, enhanced case finding, improved MDR-TB therapies, and focused political and economic commitment^44^) should be prioritized, given the potential for substantial spread in the future.

## Supplementary information

Supplementary Information Appendix

## Data Availability

The datasets generated during and/or analyzed during the current study are available from the corresponding author on reasonable request.
