# Supplementary material for: Projecting the impact of variable MDR-TB transmission efficiency on long-term epidemic trends in South Africa and Vietnam
Source: Sci Rep. 2019 Dec 2;9:18099. doi: 10.1038/s41598-019-54561-9 (PMC6889300; doi:10.1038/s41598-019-54561-9)
Supplement: Supplementary file 1 — Supplementary Information Appendix [file 41598_2019_54561_MOESM1_ESM.pdf]

SUPPLEMENTARY MATERIALS AND METHODS

for

Projecting the impact of variable MDR-TB transmission efficiency on long-term epidemic trends in South Africa and Vietnam

Phillip P. Salvatore, Emily A. Kendall, Dena Seabrook, Jessie Brown, George Durham, and David W. Dowdy

List of Supplementary Figures and Tables S.2

Supplementary Materials and Methods S.3

Model Summary S.3

- **Differential Equations** S.15
- **Sampling & Calibration** S.28
- **Bayesian Model Comparison** S.33
- **Replication of Previous Findings** S.34
- **Sensitivity Analyses** S.36

Supplementary Results S.37

- **Calibration** S.37
- **Model Projections** S.40
- **Replication of Previous Findings** S.45
- **Sensitivity Analyses** S.49

References S.56

LIST OF SUPPLEMENTARY FIGURES & TABLES

Figure S1: Transmission Efficiency Scenarios **S.7**

Figure S2: Modeled Trends in the Availability of Drug Sensitivity Testing and Second-line Treatment **S.9**

Figure S3: Modeled Trends in HIV Incidence and ART Coverage **S.14**

Table S1: TB Natural History Parameter Prior Distributions **S.30**

Table S2: HIV and TB/HIV Parameter Prior Distributions **S.31**

Table S3: MDR-TB Parameter Prior Distributions **S.32**

Table S4: Calibration Targets **S.33**

Figure S4: TB and HIV Incidence Calibration Results **S.38**

Table S5: Model Comparison with Bayes Factors **S.39**

Figure S5: Projections of the Relative Incidence of MDR-TB **S.41**

Figure S6: Projections of the MDR-TB Burden in the No Deficit Scenario **S.42**

Figure S7: Projections of the Absolute Burden of TB **S.43**

Figure S8: Projections of Acquired Drug Resistance **S.44**

Figure S9: Calibration Results of a Slower Epidemic Scenario in South Africa **S.46**

Figure S10: Projections of MDR-TB Burden in a Slower Epidemic **S.47**

Figure S11: Univariate Sensitivity Analysis – Relative MDR-TB Incidence in South Africa **S.50**

Figure S12: Univariate Sensitivity Analysis – Absolute MDR-TB Incidence in Vietnam **S.51**

Figure S13: Multivariate Sensitivity Analysis – Absolute MDR-TB Incidence **S.52**

Figure S14: TB Natural History Parameter Distributions – South Africa **S.53**

Figure S15: HIV and TB/HIV Parameter Distributions – South Africa **S.54**

Figure S16: MDR-TB Parameter Distributions – South Africa **S.55**

SUPPLEMENTARY MATERIALS AND METHODS

Model Summary

TB Natural History

Populations in our model are categorized and described by core states of TB infection and substates of resistance status (DS-TB or MDR-TB), TB treatment history (new or previously-treated), and HIV status (Fig. 1).

TB infection results from a density-dependent transmission process. Upon initial infection, populations may progress rapidly to incipient/preclinical/asymptomatic TB (hereafter referred to as early-active TB) or may develop latent infection; those latently infected may reactivate to early-active TB at a constant rate. Individuals in states of early-active TB, symptomatic active TB, ineffectively treated TB, or diagnosed-untreated TB contribute infectious person-time to transmission (with reduced infectiousness and mortality associated with early-active or ineffectively treated TB). Self-cure (with return to the susceptible state) may occur at a constant rate during early-active or active TB.

When TB patients develop fully symptomatic active TB, they may initiate first-line or (in cases of MDR-TB) second-line treatment. Upon initiating treatment, TB patients are separated into states of effective or ineffective treatment; those who complete effective treatment regimens experience culture conversion by the end of the treatment duration. (In the absence of DST, MDR-TB patients can only initiate ineffective first-line treatment). After effective treatment, TB patients may either achieve durable cure (becoming susceptible again) or may eventually relapse; after ineffective treatment, patients may either immediately re-initiate treatment or return to symptomatic active TB. During any TB treatment state, patients may be lost to follow-up; all

those lost from ineffective treatment return to active TB, while those lost from effective treatment may return to active TB or (having received a sufficiently curative treatment before default) may achieve durable cure.

MDR-TB infections may result from transmission of MDR-TB or from acquisition of drug resistance during treatment of DS-TB. MDR-TB may develop from transmission in populations who are susceptible to TB or (as a superinfection) in populations who are latently infected with DS-TB. (Similarly, populations latently infected with MDR-TB may be subsequently superinfected with DS-TB.) MDR-TB may be acquired during first-line treatment (including ineffective treatment, as well as effective treatment with future relapse).

TB treatment history influences several of the dynamics described above. TB patients with a history of first-line treatment have an increased probability of acquiring MDR-TB upon retreatment. Similarly, they have a reduced probability of receiving effective first-line treatment and a reduced probability of durable cure following effective first-line treatment. Among patients with MDR-TB who have received DST and initiated second-line therapy, those who are treated with second-line therapy unsuccessfully become treatment-ineligible (as we assume that an attempted second-line treatment may include changes in drug regimen if treatment fails to resolve symptoms).

HIV status also affects a variety of the dynamics of TB infection. HIV infected populations have higher rates of TB-independent mortality, and those with low CD4 status concurrent with active (untreated) TB experience an additional mortality associated with HIV/TB interactions. The probability of rapid TB progression upon initial infection is increased in HIV-infected populations, while the probability of TB self-cure is reduced. In populations latently infected with TB, HIV co-infection increases the rate of TB reactivation and reduces the

degree of protection against a rapidly progressing superinfection. The infectiousness of each TB disease state is somewhat lower in populations with HIV co-infection than in their HIV-uninfected counterparts. Finally, due to more rapid disease progression and more frequent encounters with the health system, active TB reaches diagnosis and treatment initiation more quickly for HIV-infected populations.

TB Transmission

TB transmission occurs as a function of time-varying transmission efficiency coefficients and the size of the infectious TB population. As previously described, we define the transmission efficiency of each strain (DS-TB vs. MDR-TB) as the number of new infections which would develop in a susceptible population from each infectious person-year contributed by prevalent infectious DS-TB or prevalent infectious MDR-TB, respectively. An initial coefficient β_S^0 is used to define the equilibrium transmission efficiency of DS-TB cases. Due to secular trends in overall TB incidence in recent years, we allow for the overall transmission of DS-TB $\beta_S(t)$ to decline at an annual geometric rate d_S after the year t_S. In Vietnam, we define t_S as the first year with data used to calibrate our epidemics ($t_S = 1996$). In South Africa, due to the HIV/TB co-epidemic over 1995-2010, we felt there was insufficient evidence for secular declines in DS-TB transmission in HIV-uninfected populations starting in the first year of survey data (2001). Instead, we assumed any such declines would not occur until 2010 ($t_S = 2010$). Therefore, we define the transmission efficiency of DS-TB as follows:

$$\beta_S(t) = \beta_S^0 \times \begin{cases} 1 & \text{if } t < t_S \\ (1 + d_S)^{t - t_S} & \text{if } t \geq t_S \end{cases} \quad (1)$$

In all MDR-TB scenarios we examined, the transmission efficiency of MDR-TB, $\beta_R(t)$, is initialized at the start of the modern MDR-TB epidemic (in the year t_R^0) with an initial value

determined by the initial transmission efficiency of DS-TB β_S^0 and a relative efficiency term E_R. In the Shrinking Efficiency Deficit scenario, the transmission efficiency of MDR-TB is allowed to increase annually (through an annual percentage increase d_R in the relative efficiency coefficient) beginning in the year t_R. (The rate of increase term d_R is set equal to zero in both the No Efficiency Deficit and Constant Efficiency Deficit scenarios, and the relative efficiency term E_R is set equal to one in the No Efficiency Deficit scenario.) Therefore, the transmission efficiency of MDR-TB is defined as:

$$\beta_R(t) = \beta_S^0 \times \begin{cases} 0 & \text{if } t < t_R^0 \\ E_R & \text{if } t_R^0 \leq t < t_R \\ E_R + (t - t_R)d_R & \text{if } t \geq t_R \end{cases} \quad (2)$$

In this way, any declines in DS-TB secular transmission efficiency (after time t_S) are not necessarily mirrored by MDR-TB, as trends in the transmission efficiency of DS-TB and MDR-TB are likely to occur through different processes (though $\beta_R(t)$ is never permitted to exceed $\beta_S(t)$). The general relationship between $\beta_S(t)$and $\beta_R(t)$ in each scenario is displayed diagrammatically in Fig. S1.

Figure S1: Transmission Efficiency Scenarios

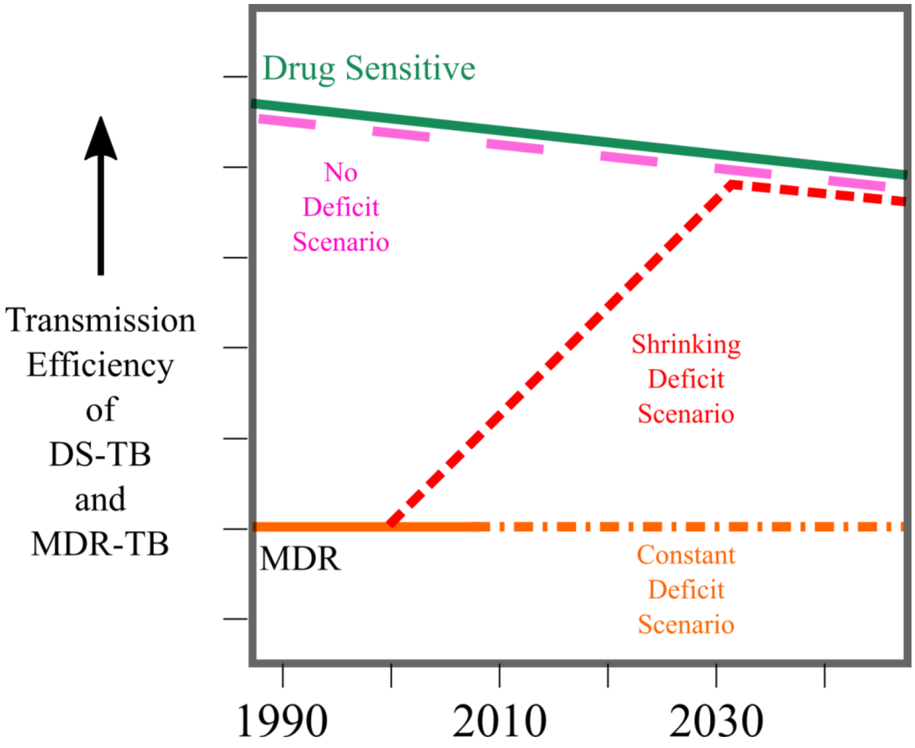

The assumed transmission efficiency (transmission events per 1,000 infectious person-years) of DS-TB over time is drawn in green; the downward slope recapitulates reductions in TB transmission efficiency due to secular trends unrelated to MDR-TB diagnosis and treatment (for example, reductions in crowding, improved socioeconomic conditions, etc.). In our three model scenarios, we assume either that the transmission efficiency of MDR-TB is at a perpetual defict compared to that of DS-TB (Constant Deficit Scenario, drawn in orange); that the transmission efficiency of MDR-TB is consistently the same as that of DS-TB (No Deficit Scenario, drawn in magenta); or that MDR-TB has lower transmission efficiency than DS-TB initially but gradually converges towards that of DS-TB over time (Shrinking Deficit Scenario, drawn in red). Years are shown for illustrative purposes; dates of MDR-TB emergence and rates of increase/decrease in transmission efficiency are sampled from defined ranges; see Sampling & Calibration for further details.

Drug Sensitivity Testing and Second-line Treatment

The probability of MDR-TB cases receiving DST and initiating second-line therapy, $DST(t)$, is defined as a country-specific time-varying quantity that depends on the availability of DST and individualized second-line regimens. We assume that DST is not widely available prior to 2006. We then separately define the probability of receiving DST during two time intervals: from 2006 to the time of Xpert® MTB/RIF adoption by national TB programs t_{Xpert} (modeled as 2011 in South Africa and 2013 in Vietnam); and from t_{Xpert} to 2016. At t_{Xpert}, we use WHO estimates of each country's provision of DST among all TB cases (data was not yet stratified by new and retreatment cases) and the proportion of detected MDR-TB cases that initiated treatment to estimate DST coverage, $\widehat{DST}(t_{Xpert})$. We modeled the availability of DST and the initiation of second-line treatment as a linear increase in $DST(t)$from $DST(2006)=0$ to the value of $\widehat{DST}(t_{Xpert})$ that we estimated from WHO data. We similarly used WHO estimates of each country's DST provision (stratified by new and retreatment cases) and treatment initiation of detected MDR-TB cases to estimate $\widehat{DST}(2016)$. Again, we modeled the availability of DST as a linear increase from the estimated $\widehat{DST}(t_{Xpert})$ to $\widehat{DST}_N(2016)$ and $\widehat{DST}(2016)$. We then assumed the linear trends of DST availability will each continue into the future beyond 2016 until eventually reaching 100% coverage. These trends are plotted graphically in Fig. S2 below, and are summarized as follows:

$$DST_N(t) = \begin{cases} 0 & \text{if } t < 2006 \\ \frac{\widehat{DST}(t_{Xpert})}{t_{Xpert}-2006}(t-2006) & \text{if } 2006 \le t < t_{Xpert} \\ \min[\,100\%\,, & \text{if } t \ge t_{Xpert} \\ \frac{\widehat{DST}_N(2016)-\widehat{DST}(t_{Xpert})}{2016-t_{Xpert}}(t-t_{Xpert}) + \widehat{DST}(t_{Xpert})] & \end{cases} \quad (3)$$

$$DST_P(t) = \begin{cases} DST_N(t) & \text{if } t < t_{Xpert} \\ \min[100\%, & \text{if } t \geq t_{Xpert} \\ \frac{\widehat{DST}_P(2016) - \widehat{DST}(t_{Xpert})}{2016 - t_{Xpert}}(t - t_{Xpert}) + \widehat{DST}(t_{Xpert})] & \end{cases} \quad (4)$$

Figure S2: Modeled Trends in the Availability of Drug Sensitivity Testing and Second-line Treatment

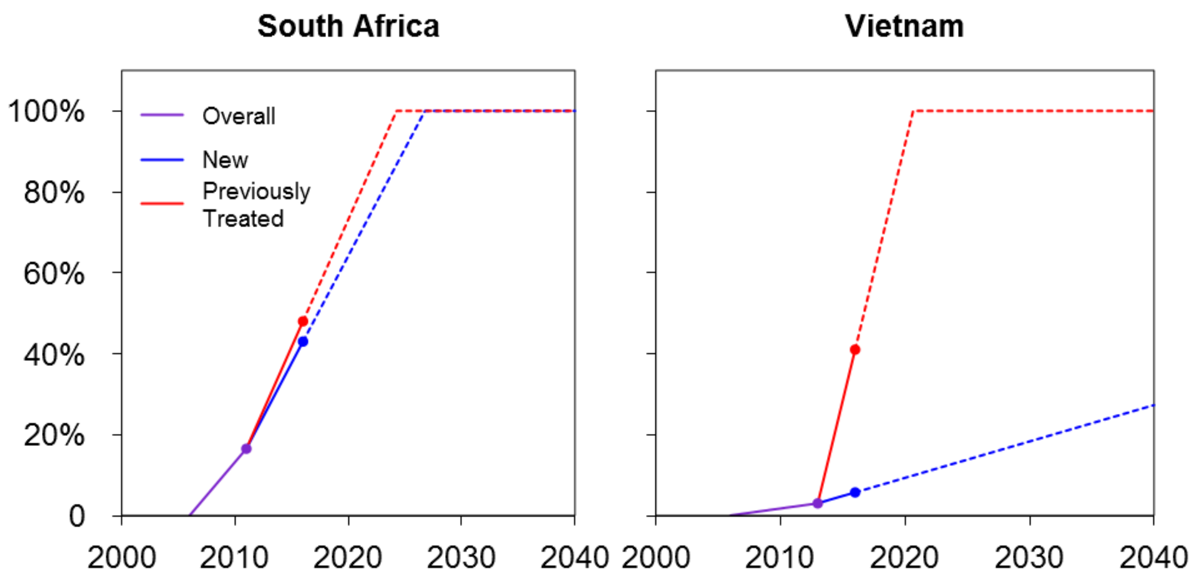

Each line represents the time-varying probability of DST for MDR-TB cases after TB diagnosis. Points represent country-specific estimates in DST availability over time drawn from WHO reports. From 2006 to 2011 in South Africa and 2013 in Vietnam, estimates of only overall (cumulative) DST were reported and therefore used for both new and previously-treated cases. Dashed lines represent current trends in DST extrapolated into the future until reaching 100% coverage.

<u>Births and Deaths</u>

We make a simplifying assumption of a steady-state population size. "Births" (entry of new 15-year olds into the population) are forced to equal cumulative deaths due to TB, HIV, and

background mortality. New adults are added to treatment-naïve susceptible or latently infected states. The proportions of new adults which enter into latent TB states are determined by the cumulative TB forces of infection over the preceding 15 years (approximated by an exponential distribution using the transmission efficiencies of TB at the midpoint 7.5 years prior and the current prevalence of infection; see differential equations below). These new latently-infected adults are divided between DS-TB and MDR-TB infections according to the ratio of each strain's approximate force of infection over the preceding 15 years.

HIV Infection and ART Initiation

Our model does not attempt to replicate the complex dynamics and partner networks which characterize transmission in HIV epidemics. To capture macro trends in the HIV epidemics of South Africa and Vietnam, we fitted our model to reported UNAIDS estimates of the prevalence of HIV among adults in yearly intervals from 1990 to 2016 (Fig. S3A below) in the following manner. At any time t_1, the prevalence of HIV infections in our fixed population of 100,000 is calculated as the sum of individuals in any HIV-infected substate *V(t)* divided by the total population size:

$$P_{HIV}(t_1) = \frac{\sum V(t_1)}{N} \qquad (5)$$

After a time interval from $[t_1,t_2)$, the prevalence of HIV survivors (without new incident infections) $P^0_{HIV}(t_2)$ would equal the prevalence at t_1 minus the number of deaths (based on the state-specific cumulative mortality rates μ^V_{All}) from all HIV-infected states during the time interval:

$$P^0_{HIV}(t_2) = P_{HIV}(t_1) - \frac{\sum V(t_1)\mu^V_{All}}{N} \qquad (6)$$

For reference, we use UNAIDS country-specific estimates of the prevalence of HIV among adults 15 years and older $\widehat{P_{HIV}}(Y)$ for the discrete time points $Y \in \{1990, 1991, \ldots, 2016\}$. The expected HIV prevalence at time t_2 is then calculated by linear interpolation between the two time points nearest in time to t_2 (the maximum Y less than t_2 and the minimum Y greater than t_2):

$$\begin{aligned}\widehat{P_{HIV}}(t_2) &= \widehat{P_{HIV}}(max(Y|Y \leq t_2)) \\ &+ \left[\frac{\widehat{P_{HIV}}(min(Y|Y \geq t_2)) - \widehat{P_{HIV}}(max(Y|Y \leq t_2))}{(min(Y|Y \geq t_2)) - (max(Y|Y \leq t_2))} \times (t_2 - (max(Y|Y \leq t_2)))\right]\end{aligned} \tag{7}$$

To reach the necessary $P_{HIV}(t_2)$ between $[t_1, t_2)$, the number of new HIV infections in this interval $I_{HIV}(t_1, t_2)$ must therefore equal the expected prevalence minus the prevalence of surviving HIV infections multiplied by the population size:

$$I_{HIV}(t_1, t_2) = (\widehat{P_{HIV}}(t_2) - P^0_{HIV}(t_2)) \times N \tag{8}$$

The rate of new HIV infections during the interval is determined by the number of new HIV infections needed and the size of currently HIV-uninfected populations in states X^U:

$$FOI_{HIV}(t_1, t_2) = \frac{I_{HIV}(t_1, t_2)}{\sum X^U(t_1)} \tag{9}$$

All incident HIV infections are modeled as transitions from an HIV-uninfected state to a state of HIV infection with a High CD4 count. After 2016, the expected rate of change in HIV prevalence is assumed to equal that of 2015-2016. At no time is the rate of new HIV infections allowed to become negative.

Prior to 2004, we assume that antiretroviral therapy (ART) is not widely available. Between 1990 and 2004, individuals in High CD4 substates gradually progress to Low CD4 substates where they remain until death. Between 2004 and 2010, we assume that ART is available only to those with Low CD4 counts. During this time, the time-varying rate of ART

initiation is derived such that the proportion of HIV-infected patients receiving ART are consistent with UNAIDS estimates (Fig. S3B below).

The rate of ART initiation is fitted to UNAIDS estimates in a similar manner used to fit HIV prevalence. We define the proportion of HIV-infected individuals receiving ART at any time *A(t)* as equal to the cumulative size of ART substates X^A divided by the cumulative size of all HIV-infected substates *V*:

$$A(t) = \frac{\sum X^A(t)}{\sum V(t)} \tag{10}$$

After a time interval from $[t_1,t_2)$, the number of survivors *continuing* to receive ART $X^{A,0}(t_2)$ would equal the number receiving ART at t_1 minus the number of deaths (based on the state-specific cumulative mortality rates μ^A_{All}) from all ART substates during the time interval:

$$\sum X^{A,0}(t_2) = \sum X^A(t_1)(1-\mu^A_{All}) \tag{11}$$

The total ART coverage (continued and newly initiated) expected at time t_2 is again fitted to UNAIDS estimates. Based on the shape of UNAIDS estimates of this quantity (Fig. S3B), we used nonlinear (weighted) least squares regression to fit a sigmoid function (assuming ART coverage approaches a future asymptote A_{max} at 60%) of the form:

$$\hat{A}\,(t) = \frac{A_{max}}{1+e^{-bt-c}} \tag{12}$$

To reach this new level of ART coverage, the number of new ART initiations during the interval $I_{ART}(t_1,t_2)$ can be calculated as the number of total individuals receiving ART at t_2 minus the number *continuing* to receive ART:

$$I_{ART}(t_1,t_2) = \left(\hat{A}\,(t_2)\sum V(t_2)\right) - \sum X^{A,0}(t_2) \tag{13}$$

The time-varying rate of ART initiation $\Omega(t_1,t_2)$ is derived according to the number of required new patients initiating ART $I_{ART}(t_1,t_2)$ and the population eligible to receive ART at time t_2. Between 2004 and 2010, all new ART initiation is restricted to Low CD4 populations X^L.

After 2010, we allow for ART initiation to occur in both Low CD4 and High CD4 populations X^H to reflect improved access to HIV care and changing national guidelines for ART provision. However, ART initiation in High CD4 populations occurs at a lower rate than initiation in Low CD4 populations, according to an initiation coefficient k_H (that is, for every one High CD4 patient, there are only k_H High CD4 patients *eligible* for ART). Therefore, the time-varying rate of ART initiation is defined as follows:

$$\Omega(t_1, t_2) = \begin{cases} 0 & \text{if } t_2 \leq 2006 \\ \frac{I_{ART}(t_1,t_2)}{\sum X^L(t_2)} & \text{if } 2006 < t_2 \leq 2010 \\ \frac{I_{ART}(t_1,t_2)}{\sum (X^L(t_2)+k_H X^H(t_2))} & \text{if } t_2 > 2010 \end{cases} \quad (14)$$

Finally, among HIV-infected patients receiving TB treatment, this rate of ART initiation is increased by a factor k_{tb} to reflect increased HIV screening in patients diagnosed with TB. Therefore, the total rate of ART initiation in TB treatment states equals $k_{tb}\Omega(t_1, t_1)$.

Figure S3: Modeled Trends in HIV Incidence and ART Coverage

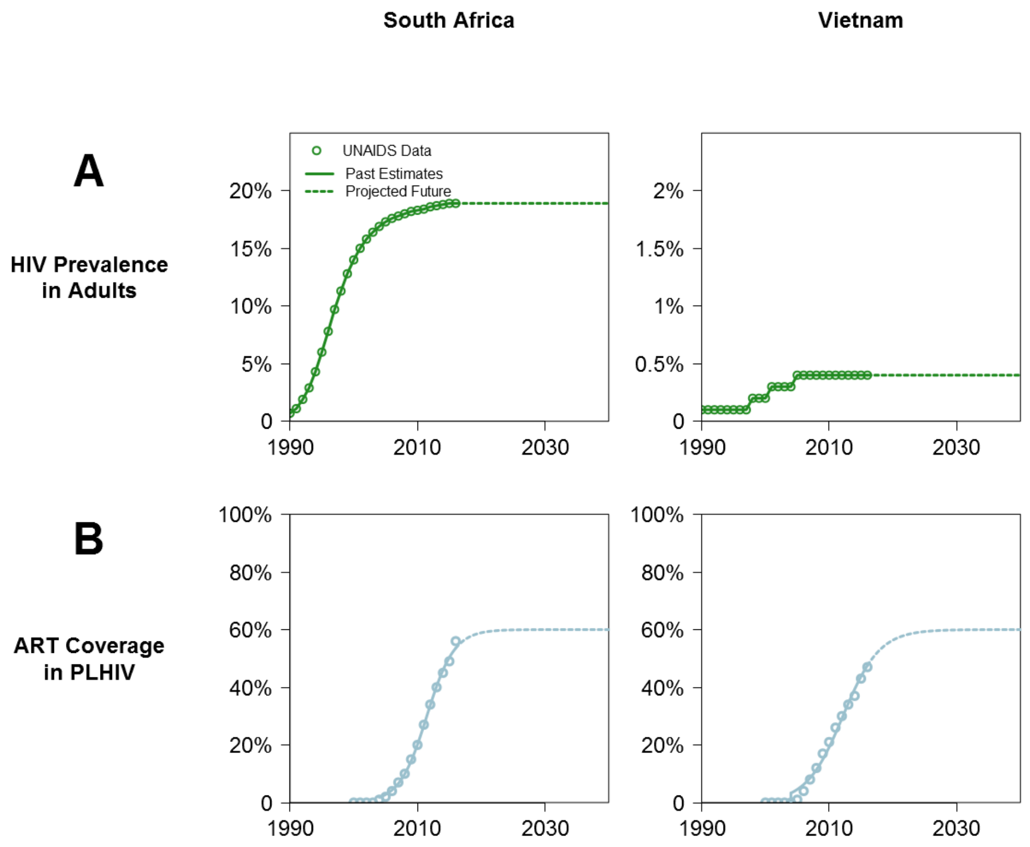

Plots present country-specific trends in HIV prevalence or ART coverage. (A) The prevalence of HIV among adults 15 years and older. Points represent UNAIDS estimates of prevalence at yearly intervals, while lines represent our interpolation/extrapolation of incidence rates from those data. (B) The proportion of persons living with HIV currently receiving ART. Points represent ART coverage from UNAIDS estimates. These estimates were used to fit sigmoid functions with ART coverage approaching a maximum of 60%. Lines represent the fitted functions which were used to model ART coverage. Dashed lines represent current trends extrapolated into the future.

Differential Equations

Notation and Conventions

Core TB states are defined according to the following abbreviations:

S = susceptible

L = latently infected

E = early-active TB (incipient TB, not yet seeking care)

A = symptomatic active TB

B_{1e} = on effective first-line treatment (leading to culture conversion)

B_{1i} = on ineffective first-line treatment (remaining infectious)

W = post-treatment, will relapse

B_{2e1} = on effective second-line treatment (the initial six months of MDR-TB treatment, remaining partially infectious)

B_{2e2} = on effective second-line treatment (the continuation phase of MDR-TB treatment, leading to culture conversion after 14 months)

B_{2i} = on ineffective second-line treatment (remaining infectious)

Z = post second-line treatment, will relapse

F = post second-line treatment, active MDR-TB

C = diagnosed untreated MDR-TB

X = any state above

I = any infectious state above $\{E, A, B_{1i}, B_{2e1}, B_{2i}, F, C\}$

G = any diagnosis/treatment state above $\{B_{1e}, B_{1i}, B_{2e1}, B_{2e2}, B_{2i}, C\}$

Populations in any of the above core TB states are additionally categorized using the following subscripts and superscripts (some combinations may be null):

T = treatment status (a value of N indicates TB treatment-naïve; a value of P indicates previously-treated for TB)

D = drug sensitivity status (a value of S indicates DS-TB infections; a value of R indicates MDR-TB infections)

V = HIV status (a value of U indicates HIV-uninfected; a value of H indicates HIV-infected with High CD4 counts; a value of L indicates a HIV-infected with Low CD4 counts; a value of A indicates HIV-infected and receiving ART)

As described above, several rates and probabilities of core TB states are modified by TB treatment history and HIV status. Unless otherwise noted, parameter values with a superscript of naught (“0”) represent the rate/probability in HIV-negative, treatment-naïve patients; values with a superscript of $V \in \{U, H, L, A\}$ reflect the multiplicative factor associated with each respective HIV substate; and values with a superscript of $T \in \{N, P\}$ reflect the multiplicative factor associated with each respective category of TB treatment history. Thus, if ρ^0 represents the probability of rapid progression in HIV-uninfected populations, then $\rho^0\rho^H$ represents the probability of rapid progression in HIV-infected populations with a High CD4 count. (Multiplicative factors with superscripts of *U* or *N* are set equal to one; thus $\rho^0\rho^U = \rho^0$.)

Populations Susceptible to TB Infection

The total population is initiated at a size of 100,000 and forced to remain at steady-state. The size of newly added populations is forced to equal the number of deaths in the population at any time, defined by the rates of background mortality (μ^0), HIV-associated mortality (μ^V), and

active-TB associated mortality (μ$_{tb}$, reduced by factors i_X associated with early/incompletely treated active TB):

$$M(t) = \sum_{V\in\{U,H,L,A\}} \left[\sum_{X^V\in I_D^V} \left(X^V(t)(\mu^0 + \mu^V + \mu_{tb})\right) + \sum_{X^V\notin I_D^V} \left(X^V(t)(\mu^0 + \mu^V)\right) \right] \quad (15)$$

All newly added populations are divided between susceptible and latently infected states (described above). We define the forces of infection applied to individuals before entering the population at age 15 as using the approximate forces of infection distributed over the preceding 7.5 years:

$$F\hat{O}I_D\,(t - 7.5) = \sum_{V\in\{U,H,L,A\}} \left[i^V \sum_{X^V\in\{I_D^V\}} (X^V(t) i_X \beta_D(t - 7.5)) \right] \quad (16)$$

These approximate forces of infection are used to determine the number of new 15-year olds entering the population as susceptible to TB infection. Newly added susceptible populations S_N occur to replace deaths (excluding those latently infected) and those who self-cure (according to the rate of self-cure (ν^0)) without treatment. Transitions from the susceptible state occur due to new infection (dependent on the time-varying forces of infection ($FOI_D(t)$), described below) and death:

$$\frac{dS_N^U}{dt} = M(t)e^{-\sum_{D\in\{S,R\}}\left(F\hat{O}I_D(t-7.5)\right)} + \nu^0 \left(\sum_{D\in\{S,R\}} E_{ND}^U(t) + A_{ND}^U(t) \right) - S_N^U(t) \left(\mu^0 + \sum_{D\in\{S,R\}} FOI_D(t) \right) \quad (17)$$

All newly susceptible 15-year olds are modeled as initially HIV-uninfected (ignoring contributions from perinatal infection). In HIV-infected populations, new susceptible populations occur only through TB self-cure:

For V $\in \{H, L, A\}$:

$$\frac{dS_N^V}{dt} = \nu^0 \nu^V \left(\sum_{D\in\{S,R\}} E_{ND}^V(t) + A_{ND}^V(t) \right) - S_N^V(t) \left(\mu^0 + \mu^V + \sum_{D\in\{S,R\}} FOI_D(t) \right) \quad (18)$$

Transitions into the previously-treated susceptible state S_P occur as the result of self-cure ($\nu^0\nu^V$) of previously-treated patients with early-active E_P (due to new infection after previous cure) or active TB A_P (after failed treatment or new infection after previous cure).

Effective first-line treatment B1eS can result in also durable cure (returning to the susceptible state S_P) after treatment has been completed (duration $\tau_{t1} = 6$ months) without the acquisition of MDR during treatment (based on the probability of MDR acquisition during treatment $\alpha^0\alpha^T$) in two ways: a) treatment is completed without loss to follow-up (based on the probability of loss during first-line treatment δ_1) and without subsequent relapse (based on the probability of relapse among those with DS-TB who complete first-line treatment $\omega_1^0\omega_1^T$); or b) treatment is not completed due to loss to follow-up but after a sufficiently curative treatment has been received (based on the proportion of those lost during first-line treatment who remain culture positive at the time of loss η_1). Durable cure can also be achieved after the continuation phase of effective second-line B_{2e2} treatment (duration $\tau_{22} = 14$ months) if relapse does not occur (probability $1 - \omega_2^0$). Transitions out of the previously-treated susceptible state occur due to death or new infection.

$$\begin{aligned}\frac{dS_P^V}{dt} &= \nu^0\nu^V\left(\sum_{D\in\{S,R\}} E_{PD}^V(t) + A_{PD}^V(t)\right)\\ &+\sum^{T\in\{S,P\}}\left[B_{1eST}^V(t)\frac{1-\alpha^0\alpha^T}{\tau_{t1}}((1-\delta_1)(1-\omega_1^0\omega_1^T)+\delta_1(1-\eta_1))\right]\\ &+B_{2e2}^V(t)(1-\omega_2)/\tau_{22} - S_P^V(t)\left(\mu^0+\mu^V+\sum_{D\in\{S,R\}} FOI_D(t)\right)\end{aligned} \tag{19}$$

Transmission of TB Infection

New latently infected populations L_N may result from the addition of previously-infected 15-year olds (all assumed to be HIV-uninfected), new infections, or superinfection of already latently-infected populations. The total number of new 15-year olds is equal to the number of deaths $M(t)$ as above, and the proportion that enters with a previous latent infection is determined

by an exponential distribution of the cumulative estimated force of infection over 15 years ($\sum_{D\in\{S,R\}}\big(F\hat{O}I_D\ (t-7.5)\big)$); these latent infections are divided between DS-TB and MDR-TB weighted by the strain-specific force of infection during this period ($F\hat{O}I_D\ (t-7.5)$).

New infections from susceptible states S occur based on the current force of infection $FOI_D(t)$ and the probability that new infections do not progress rapidly to early-active TB ($1-\rho^0$). The time-varying force of infection for a given TB strain $FOI_D(t)$ describes the cumulative, state-adjusted infectious person-time contributed by all TB cases. For a given infectious state I_D^V, the population size of $I_D^V(t)$ is reduced by the relative infectiousness of the core TB state ix (associated with early-active disease or incomplete/ineffective treatment) and the relative infectiousness of the HIV substate i^V, and multiplied by the transmission efficiency $\beta_D(t)$ (defined above).

$$FOI_D(t) = \sum_{V\in\{U,H,L,A\}}\left[i^V \sum_{X^V\in\{I_D^V\}} (X^V(t) i_X \beta_D(t))\right] \qquad (20)$$

Transitions between MDR-TB and DS-TB latent states (e.g., L_{NS} to L_{NR}) occur due to exogenous superinfections which do not progress rapidly ($1-\rho^0$). Additional protection against rapid infection is afforded by an existing latent infection (based on the reduced probability of rapid progression in latently infected populations $1-\lambda^0$). The proportions of superinfections which change state (e.g., latent DS-TB to latent MDR-TB) are weighted by the strain-specific transmission efficiencies such that, if $\beta_S(t) = \beta_R(t)$, then half of reinfected latent DS-TB patients will transition to latent MDR-TB patient and vice versa.

Transitions out of the new, latently infected state occur due to background mortality, reactivation to early-active TB (based on the constant rate of reactivation (r^0)), or reinfection

followed by rapid progression (despite the protection afforded by an existing latent infection $((1-\lambda^0)\rho^0)$).

$$\begin{aligned}\frac{dL_{ND}^U(t)}{dt} &= M(t)\frac{F\hat{O}I_D(t-7.5)}{\sum_{D\in\{S,R\}}\left(F\hat{O}I_D(t-7.5)\right)}\left(1-e^{-\sum_{D\in\{S,R\}}\left(F\hat{O}I_D(t-7.5)\right)}\right)\\ &+S_N^U(1-\rho^0)FOI_D(t)\\ &+(1-\rho^0(1-\lambda^0))\left[\frac{\beta_D(t)}{\beta_D(t)+\beta_{\overline{D}}(t)}FOI_D(t)L_{N\overline{D}}^U(t)-\frac{\beta_{\overline{D}}(t)}{\beta_D(t)+\beta_{\overline{D}}(t)}FOI_{\overline{D}}(t)L_{ND}^U(t)\right]\\ &-L_{ND}^U(t)(r^0+\mu^0)-(1-\lambda^0)\rho^0FOI_D(t)\sum_{D\in\{S,R\}}(L_{ND}^U(t))\end{aligned} \tag{21}$$

As described above, all new 15-year olds are assumed to be initially uninfected. Therefore, new additions to HIV-infected latent states occur due to new infections of susceptible populations and superinfections of latent populations. Losses occur in the same manner as HIV-uninfected latent populations.

For V $\in\{H,L,A\}$:

$$\begin{aligned}\frac{dL_{ND}^V}{dt} &= S_N^V(1-\rho^0\rho^V)FOI_D(t)\\ &+(1-\rho^0\rho^V(1-\lambda^0\lambda^V))\left[\frac{\beta_D(t)}{\beta_D(t)+\beta_{\overline{D}}(t)}FOI_D(t)L_{N\overline{D}}^V(t)-\frac{\beta_{\overline{D}}(t)}{\beta_D(t)+\beta_{\overline{D}}(t)}FOI_{\overline{D}}(t)L_{ND}^V(t)\right]\\ &-L_{ND}^V(t)(r^0r^V+\mu^0+\mu^V)-(1-\lambda^0\lambda^V)\rho^0\rho^VFOI_D(t)\sum_{D\in\{S,R\}}(L_{ND}^V(t))\end{aligned} \tag{22}$$

Among previously-treated populations, latent infections L_P result from new infections of previously-treated TB susceptible populations and superinfections of previously-treated, latently infected populations in the same manner as above. (Newly added 15-year olds are assumed to have negligible previous TB treatment history and are excluded from these populations upon initial entry.)

$$\begin{aligned}\frac{dL_{PD}^V(t)}{dt} &= S_P^V(1-\rho^0\rho^V)FOI_D(t)\\ &+(1-\rho^0\rho^V(1-\lambda^0\lambda^V))\left[\frac{\beta_D(t)}{\beta_D(t)+\beta_{\overline{D}}(t)}FOI_D(t)L_{P\overline{D}}^V(t)-\frac{\beta_{\overline{D}}(t)}{\beta_D(t)+\beta_{\overline{D}}(t)}FOI_{\overline{D}}(t)L_{PD}^V(t)\right]\\ &-L_{PD}^V(t)(r^0r^V+\mu^0+\mu^V)-(1-\lambda^0\lambda^V)\rho^0\rho^VFOI_D(t)\sum_{D\in\{S,R\}}(L_{PD}^V(t))\end{aligned} \tag{23}$$

Progression into early-active states E occur from a) a susceptible state S according to the force of infection and the proportion of infections which progress rapidly $(\rho^0\rho^V)$; b) from a latent

state L due to reactivation of endogenous infection according to the rate of reactivation ($r^0 r^V$); or c) from rapid progression of a recent, exogenous superinfection according to the proportion of infections which progress rapidly, reduced by the protection afforded by an existing latent infection ($(1-\lambda^0\lambda^V)\rho^0\rho^V$). Transitions from the early-active state occur due to death, spontaneous resolution, or progression to active TB.

$$\begin{aligned}\frac{dE_{TD}^V}{dt} &= S_T^V(t)\rho^0\rho^V FOI_D(t) + L_{TD}^V r^0 r^V + (1-\lambda^0\lambda^V)\rho^0\rho^V FOI_D(t)\sum_{D\in\{S,R\}}(L_{TD}^V) \\ &-E_{TD}^V(t)(1/a + \nu^0\nu^V + \mu^0 + \mu^V + i_E\mu_{tb})\end{aligned} \quad (24)$$

<u>Active TB</u>

Transitions into the symptomatic, active TB states A occur from the progression of early-active TB E according to the duration of early-active TB a. Transitions from the active disease state occur due to death, spontaneous resolution, or treatment initiation.

$$\frac{dA_{ND}^V}{dt} = aE_{ND}^V(t) - A_{ND}^V\left(\frac{1}{x^0x^V} + \nu^0\nu^V + \mu^0 + \mu^V + \mu_{tb}\right) \quad (25)$$

In those previously treated, active disease A_P may additionally result from: a) relapse among those who will relapse W after the mean time to relapse (τ_ω); b) loss to follow-up (δ_1) during ineffective first-line treatment B_{1i} ; c) loss to follow-up during effective treatment B_{1e} provided that culture conversion has not yet occurred ($\delta_1\eta_1$); or d) failing ineffective first-line therapy provided that treatment has been completed without loss to follow-up $(1-\delta_1)$ after treatment duration $\tau_{t1} = 6$ months and that care is not immediately reinitiated $(1-\gamma)$.

$$\begin{aligned}\frac{dA_{PS}^V}{dt} &= aE_{PS}^V(t) + W_S^V(t)(1/\tau_\omega) \\ &+\frac{1}{\tau_{t1}}\sum_{T\in\{N,P\}}\left[(1-\alpha^0\alpha^T)\left(B_{1eST}^V(t)\delta_1\eta_1 + B_{1iST}^V(t)(\delta_1 + (1-\delta_1)(1-\gamma))\right)\right] \\ &-A_{PS}^V(t)\left(\frac{1}{x^0x^V} + \nu^0\nu^V + \mu^0 + \mu^V + \mu_{tb}\right)\end{aligned} \quad (26)$$

In those with MDR-TB, active disease A_{PR} may also result from the new acquisition of drug resistance during ineffective first-line therapy B_{1iS} based on the probability of MDR

acquisition during first-line treatment $\alpha^0\alpha^T$. (Patients with preexisting MDR-TB only initiate ineffective first-line treatment B_{1iR}, described below.)

$$\begin{aligned}\frac{dA_{PR}^V}{dt} &= aE_{PR}^V + W_R^V(t)(1/\tau_\omega)\\ &+\frac{1}{\tau_{t1}}\sum_{T\in\{N,P\}}\left(\alpha^0\alpha^T B_{1iST}^V + B_{1iTR}^V(t)\right)(\delta_1 + (1-\delta_1)(1-\gamma))\\ &-A_{PR}^V(t)\left(\frac{1}{x^0x^V} + \nu^0\nu^V + \mu^0 + \mu^V + \mu_{tb}\right)\end{aligned} \tag{27}$$

Among those with MDR-TB who have been diagnosed and received DST, some may be lost before linkage to appropriate second-line therapy while others may not receive second-line therapy based on the specific drug resistance pattern (e.g., extensively drug resistant TB) and available drug regimens. These patients enter in the diagnosed active but untreated MDR-TB state *C*. These patients must first receive DST (with probability $DST_T(t)$, described above) at the time of diagnosis or re-evaluation (after a delay in diagnosis – duration x^0x^V – or at the completion of a failed first-line regimen – duration $\tau_{t1} = 6$ months). MDR-TB patients eligible to receive DST are those with active, untreated TB A_R; those with pre-existing MDR-TB at the completion of an ineffective first-line regimen; or those with DS-TB who acquire MDR-TB during first-line therapy B_{1eS} with (probability $\alpha^0\alpha^T$). Those receiving a first-line therapy are eligible for a DST re-evaluation provided they complete the first-line regimen without loss to follow-up $(1-\delta_1)$ and reinitiate care immediately upon completion (γ). After receiving DST, these patients are lost/declined second-line treatment with probability *b*. Losses from C occur due to death.

$$\begin{aligned}\frac{dC_R^V}{dt} &= b\sum_{T\in\{N,P\}}\left[DST_T(t)\left(\frac{1}{x^0x^V}A_{RT}^V(t) + \frac{\gamma(1-\delta_1)}{\tau_{t1}}\left(B_{1iRT}^V(t) + \alpha^0\alpha^T B_{1eST}^V(t)\right)\right)\right]\\ &-C_R^V(t)(\mu^0 + \mu^V + \mu_{tb})\end{aligned} \tag{28}$$

MDR-TB patients who have initiated second-line treatment without successful completion enter the failed second-line therapy state *F*. These transitions include those who

initiate successful treatment B_{2e1} but are subsequently lost to follow-up with probability δ_2; those who experience treatment failure B_{2i} (due to ineffective second-line therapy after duration $\tau_{t21} =$ 6 months); or those complete treatment but subsequently relapse Z_R (after a mean time to relapse τ_ω). Losses from F occur due to death.

$$\frac{dF_R^V}{dt} = \delta_2 B_{2e1}^V(t) + \frac{1}{\tau_{t21}} B_{2i}(t) + \frac{1}{\tau_\omega} Z_R^V(t) - F_R^V(t)(\mu^0 + \mu^V + \mu_{tb}) \quad (29)$$

TB Treatment

Upon the initiation of treatment, TB patients are separated between effective and ineffective TB treatment states based on the probability of culture conversion among those who complete treatment ($\sigma_1^0\sigma_1^T$ for first-line therapy and σ_2 for second-line therapy).

Transitions into the effective first-line therapy B_{1e} occur from the symptomatic active TB states A according after a mean delay before diagnosis (duration x^0x^V). Transitions out of B_{1e} occur due to death or ending treatment.

$$\frac{dB_{1eNS}^V}{dt} = A_{NS}^V(t)\frac{\sigma_1^0\sigma_1^N}{x^0x^V} - B_{1eNS}^V(t)(\mu^0 + \mu^V + 1/\tau_{t1}) \quad (30)$$

Among those previously treated, effective first-line therapy B_{1eP} may also be initiated following the failure of a previous first-line treatment regimen B_{1i}. This only occurs provided the previous regimen was completed (after duration $\tau_{t1} = 6$ months) without loss to follow-up ($1 - \delta_1$), that retreatment is started immediately upon completion of the previous regiment (γ), and that MDR-TB was not acquired during the previous regimen ($1 - \alpha^0\alpha^T$)).

$$\begin{aligned}\frac{dB_{ePS}^V}{dt} &= \sigma_1^0\sigma_1^P\left[\frac{1}{x^0x^V}A_{PS}^V(t) + \frac{1}{\tau_{t1}}\sum_{T\in\{N,P\}}\left(B_{1iST}^V(t)(1-\alpha^0\alpha^T)(1-\delta_1)\gamma\right)\right] \\ &\quad - B_{1ePS}^V(t)(\mu^0 + \mu^V + 1/\tau_{t1})\end{aligned} \quad (31)$$

Among DS-TB patients, transitions into the state of ineffective first-line therapy B_{1iS} occur from the same sources of effective first-line therapy above (according to the probability that treatment will not be successful $1 - \sigma_1^0\sigma_1^N$).

$$\frac{dB_{1iNS}^V}{dt} = A_{NS}^V(t)\frac{1-\sigma_1^0\sigma_1^N}{x^0x^V} - B_{1iNS}^V(t)(\mu^0 + \mu^v + 1/\tau_{t1}) \tag{32}$$

$$\begin{aligned}\frac{dB_{iPS}^V}{dt} &= (1-\sigma_1^0\sigma_1^P)\left[\frac{1}{x^0x^V}A_{PS}^V(t) + \frac{1}{\tau_{t1}}\sum_{T\in\{N,P\}}\left(B_{1iST}^V(t)(1-\alpha^0\alpha^T)(1-\delta_1)\gamma\right)\right] \\ &\quad -B_{1ePS}^V(t)(\mu^0 + \mu^V + 1/\tau_{t1})\end{aligned} \tag{33}$$

In those with MDR-TB, all first-line therapy is ineffective. Transitions into B_{1iR} occur from treatment initiation or re-initiation based on the proportion of cases which do not receive DST $(1 - DST_T(t))$.

$$\frac{dB_{1iNR}^V}{dt} = A_{NR}^V(t)\frac{1-DST_N}{x^0x^V} - B_{1iNR}^V(t)(\mu^0 + \mu^v + 1/\tau_{t1}) \tag{34}$$

$$\begin{aligned}\frac{dB_{iPR}^V}{dt} &= A_{PR}^V(t)\frac{1-DST_P(t)}{x^0x^V} + \frac{1}{\tau_{t1}}\sum_{T\in\{N,P\}}\left(B_{1iRT}^V(t)(1-DST_T(t))(1-\alpha^0\alpha^T)(1-\delta_1)\gamma\right) \\ &\quad -B_{1iPR}^V(t)(\mu^0 + \mu^V + 1/\tau_{t1})\end{aligned} \tag{35}$$

As with first-line therapy, TB patients initiating second-line therapy are separated between effective and ineffective TB treatment states based on the probability of culture conversion among those who complete treatment (σ_2). Those who enter the effective second-line treatment state begin with the initiation phase B_{2e1} (duration $\tau_{t21} = 6$ months) followed by the continuation phase B_{2e2} (duration $\tau_{t21} = 14$ months) upon completion of the initiation phase. Those who enter the ineffective second-line treatment state B_{2i} will only receive treatment for a duration of τ_{t21} before treatment is withdrawn.

Transitions into the initiation phase of effective second-line therapy B_{2e1} occur as the result of: a) symptomatic active MDR-TB which, after a delay (duration x^0x^V) before diagnosis, has received DST and was not lost before treatment initiation ((DST_T) $(1 - b)$); b) failure of ineffective first-line treatment for pre-existing MDR-TB B_{1iR} or DS-TB (B_{1iS}) which

subsequently acquired MDR-TB (with probability $\alpha^0\alpha^T$). In the case of failure, patients must have completed treatment (duration $\tau_{t1} = 6$ months) without loss to follow-up followed by immediate retreatment$(1-\delta_1)\gamma$. Transitions from *B2e1* occur due to death or finishing the initiation phase.

$$\frac{dB_{2e1}^V}{dt} = \sigma_2(1-b)\sum_{T\in\{N,P\}}\left(\frac{DST_T(t)}{x^0x^V}A_{TR}^V(t) + \frac{DST_T(t)}{\tau_{t1}}(1-\delta_1)\gamma(\alpha^0\alpha^T B_{1iST}^V(t) + B_{1iRT}^v(t))\right) \\ -B_{2e1}^V(t)(\mu^0+\mu^V+i_{B_{2e1}}\mu_{tb}+1/\tau_{t21}) \quad (36)$$

Transitions into ineffective second-line therapy for MDR-TB *B2i* occur from the same states as above.

$$\frac{dB_{2i}^V}{dt} = (1-\sigma_2)(1-b)\sum^{T\in\{N,P\}}\left(\frac{DST_T(t)}{x^0x^V}A_{TR}^V(t) + \frac{DST_T(t)}{\tau_{t1}}(1-\delta_1)\gamma(\alpha^0\alpha^T B_{1iST}^V(t) + B_{1iRT}^v(t))\right) \\ -B_{2i}^V(t)(\mu^0+\mu^V+i_{B_{2i}}\mu_{tb}+1/\tau_{t21}) \quad (37)$$

Transitions into the continuation phase of effective second-line therapy for MDR-TB *B2e2* occur only after completion of the initiation phase *B2e1* (duration $\tau_{t21} = 6$ months), provided patients were not lost to follow-up $(1-\delta_2)$. Transitions from this state occur due to death or finishing treatment (duration τ_{t22}= 14 months).

$$\frac{dB_{2e2}^V}{dt} = B_{2e1}^V(t)\frac{1-\delta_2}{\tau_{t21}} - B_{2e2}^V(t)(\mu^0+\mu^V+i_{B_{2e2}}\mu_{tb}+1/\tau_{t22}) \quad (38)$$

After Treatment

Among those who complete effective first-line therapy *B1e* (culture negative at the end of treatment), a proportion ω_2 will eventually relapse to active TB *A*. Between the completion of first-line treatment and the time of relapse, these populations occupy an asymptomatic state *W* for a mean duration of τ_ω. Transitions into *W* occur following completion of effective first-line therapy (after duration τ_{t1}) with or without the acquisition of MDR during treatment (based on the probability of acquisition $\alpha^0\alpha^T$) leading to eventual relapse with DS-TB (W_S) or MDR-TB (W_R), respectively. Losses from these states occur due to death or relapse.

$$\frac{dW_S^V}{dt} = \sum_{T\in\{N,P\}} \left(\frac{\omega_T}{\tau_{t1}}(1-\alpha^0\alpha^T)B_{1eST}^V(t)\right) - W_S^V(t)(\mu^0 + \mu^V + 1/\tau_\omega) \quad (39)$$

$$\frac{dW_R^V}{dt} = \sum^{T\in\{N,P\}} \left(\frac{\omega_T}{\tau_{t1}}\alpha^0\alpha^T B_{1eST}^V(t)\right) - W_R^V(t)(\mu^0 + \mu^V + 1/\tau_\omega) \quad (40)$$

Among those who complete effective second-line therapy B_{2e2} (culture negative at the end of treatment), a proportion ω_2 will eventually relapse to chronic (untreated) MDR-TB *C*. Between the completion of second-line treatment and the time of relapse, these populations occupy an asymptomatic state *Z* for a mean duration of τ_ω. This population differs from that of W_R through a history of attempted second-line therapy; afterwards, those who relapse from *Z* will be ineligible for retreatment with second-line therapy. Losses from this state occur due to death or relapse.

$$\frac{dZ_R^V}{dt} = \frac{\omega_2}{\tau_{t22}} B_{2e2}^V(t) - Z_R^V(t)(\mu^0 + \mu^V + 1/\tau_\omega) \quad (41)$$

<u>HIV Infection</u>

In addition to the rates of change across states of TB infection (and births/deaths) described above, transitions between HIV substates occur due to initial HIV infection (Uninfected to High CD4), gradual immunosuppression (High CD4 to Low CD4), or ART initiation. Interactions between TB state and HIV substate influence HIV mortality (excess mortality rates in Low CD4 patients with active, untreated TB; see Table S2.1) and the rate of ART initiation (HIV patients receiving TB treatment initiate ART at an increased rate).

New High CD4 infections X^H occur based on the fitted HIV force of infection (described above); prior to 2010, transitions from the High CD4 substate occur only due to immunosuppression or death. (In our notating convention, HIV-associated mortality is presented in differential equations of TB transitions above and is therefore not duplicated here.) After 2010, transitions from the High CD4 substate may occur due to death or ART initiation.

New Low CD4 infections X^L occur solely due to immunosuppression based on the rate of CD4 decline, with an approximate duration of $1/\phi_H$ between initial infection and decline below 250 CD4 cells/mL. Prior to 2006, transitions from the Low CD4 substate occur solely due to death; after 2006, transitions from the Low CD4 substate may occur due to death or ART initiation.

HIV-infected populations are assumed to be unable to access ART before at least 2006. After 2006, Low CD4 populations become eligible for ART at a time-varying rate $\Omega(t)$ described in detail above. After 2010, High CD4 populations also become eligible for ART at a rate lower than that among Low CD4 populations (determined by the relative proportion of new ART initiations from patients with High CD4 counts k_H). For populations in any TB treatment state G, ART initiation occurs at an amplified rate ($k_{tb}\Omega(t)$). These dynamics are summarized as follows:

For X $\notin G$:
$$\frac{dX^H}{dt} = \begin{cases} FOI_{HIV}(t)X^U(t) - X^H(t)\phi_H & \text{if } t < 2010 \\ FOI_{HIV}(t)X^U(t) - X^H(t)(k_H\Omega(t) + \phi_H) & \text{if } t \geq 2010 \end{cases}$$

$$\frac{dX^L}{dt} = \begin{cases} X^H(t)\phi_H & \text{if } t < 2006 \\ X^H(t)\phi_H - X^L(t)\,\Omega(t) & \text{if } t \geq 2006 \end{cases} \qquad (42)$$

For X $\in G$:
$$\frac{dX^H}{dt} = \begin{cases} FOI_{HIV}(t)X^U(t) - X^H(t)\phi_H & \text{if } t < 2010 \\ FOI_{HIV}(t)X^U(t) - X^H(t)(k_{tb}k_H\Omega(t) + \phi_H) & \text{if } t \geq 2010 \end{cases}$$

$$\frac{dX^L}{dt} = \begin{cases} X^H(t)\phi_H & \text{if } t < 2006 \\ X^H(t)\phi_H - X^L(t)(k_{tb}\Omega(t)) & \text{if } t \geq 2006 \end{cases} \qquad (43)$$

$$\frac{dX^A}{dt} = \begin{cases} 0 & \text{if } t < 2006 \\ \Omega(t)\left[\sum_{X\notin G}(X^L(t)) + k_{tb}\sum_{X\in G}(X^L(t))\right] & \text{if } 2006 \leq t < 2010 \\ \Omega(t)\left[\sum_{X\notin G}(X^L(t) + k_H X^H(t)) + k_t b\sum_{X\in G}(X^L(t) + k_H X^H(t))\right] & \text{if } t \geq 2010 \end{cases}$$
(44)

Sampling & Calibration

To enrich our projections using simulated TB epidemics which were most consistent with empirical estimates of TB epidemics in South Africa and Vietnam, we implemented a two-stage semi-Bayesian Sampling/Importance-Resampling algorithm.[1] In the first stage of calibration, parameter sets were composed of a single value for each DS-TB or HIV parameter (drawn using Latin hypercube sampling [2], Tables S1-S2 below) while values for MDR-TB parameters (Table S3) were set to zero. Each parameter set was then assigned a likelihood value based on the DS-TB/HIV epidemic the set produced. Joint likelihoods were defined by the each epidemic's pseudo-likelihood of absolute TB incidence (per 100,000) in 2005, 2010, and 2015 (from WHO country estimates [3]) as well as the proportion of incident TB cases infected with HIV. Targets for HIV calibration were taken from a 2012 national TB drug resistance survey in South Africa [4]; comparable data from surveys in Vietnam were unavailable, thus the most recent WHO estimate of incident HIV-infected TB cases (in 2015) was used. Targets were modeled as independent beta distributions with bounds set equal to the 95% confidence limits of estimated TB incidence; bounds were set equal to 85% and 115% of the HIV point estimate. (All calibration targets are presented in Table S4 below.) The product of each simulation's pseudo-likelihoods was used as the joint likelihood of the corresponding DS-TB parameter set. Each parameter set was weighted then weighted according to the quotient of the set's joint likelihood L(θ_i ; y) and the cumulative likelihood of all sets:

$$q_i = \frac{L(\theta_i;y)}{\sum_{j=1}^{n} L(\theta_j;y)} \qquad (45)$$

DS-TB parameter sets were then resampled (with replacement) proportional to the weights assigned above. In the second stage of calibration, each resampled DS-TB parameter set was paired with a new MDR-TB parameter set (again drawn using Latin hypercube sampling,

Table S2.3 below). Each new parameter set was then used to simulate a DS-TB epidemic to a time equal to the value of the MDR-TB parameter t_R^0. After this time, MDR-TB epidemics were simulated forward to 2040. Each epidemic was then assigned a new joint likelihood defined by the epidemics pseudo-likelihood of the proportion of recent TB diagnoses with MDR-TB among new and previously-treated cases.

Table S1: TB Natural History Parameter Prior Distributions

	Description	Median	Sampling Range†	Distribution	References
μ^0	Baseline mortality rate for ages 15+ (per year)	0.017	(0.015-0.018)	Lognormal	5
μ_{tb}	Added mortality rate of untreated symptomatic TB in HIV- populations (per year)	0.15	(0.07-0.3)	Lognormal	3,6
ρ^0	Probability of rapid progression after initial TB infection in HIV- populations	0.14	(0.07-0.25)	Logit-normal	7
λ^0	Protection by latent infection: reduction in rapid progression after second infection event in HIV-populations*	0.5	(0.1-0.9)	Logit-normal	7,8
r^0	Reactivation rate, latent to early (asymptomatic) active TB in HIV- populations (per year)	0.001	(0.0005-0.002)	Lognormal	8–11
a	Duration of asymptomatic (preclinical) TB if no death or spontaneous resolution (years)	0.6	(0.36-1.0)	Lognormal	12,13
i_E	Infectiousness and mortality of asymptomatic (preclinical) TB, relative to symptomatic TB in HIV- populations	0.22	(0.1-0.4)	Logit-normal	14,15
ν^0	Rate of spontaneous resolution of untreated active TB in HIV-populations (per year)	0.13	(0.08-0.2)	Lognormal	6
x^0	Time to TB diagnosis and treatment initiation (with pretreatment loss to follow up incorporated for DS-TB) in HIV- populations (years)	1	(0.67-1.5)	Lognormal	3,12,16
σ_1^0	First-line treatment success (fraction of new DS-TB patients who will successfully complete treatment if adherent; includes those who may relapse with or without acquired resistance)	0.98	(0.96-0.99)	Logit-normal	3,17–19
σ_1^P	Reduction in first-line treatment success for previously treated DS-TB patients (multiplicative factor)	0.95	(0.9-1.0)	Uniform	3,20
ω_1^0	Relapse risk after first-line therapy (if no acquired drug resistance), new DS-TB patients	0.04	(0.026-0.06)	Logit-normal	21,22
ω_1^P	Increase in relapse risk after first-line therapy, retreatment DS-TB patients (multiplicative factor)	2	(1-3)	Uniform	20,21
τ_ω	Median time to relapse, among patients who will relapse (years)	1.5	(0.9-2.5)	Lognormal	21
δ_1	Probability of loss to follow up during first-line therapy	0.06	(0.035-0.10)	Logit-normal	3
η_1	Probability of returning to active TB after loss to follow-up during effective first-line treatment of DS-TB	0.4	(0.16-0.7)	Logit-normal	23,24
Γ	Probability of immediate retreatment after DS-TB treatment failure	0.92	(0.85-1.0)	Uniform	Model Assumption
i_A	Infectiousness and mortality of TB on ineffective treatment, relative to untreated active TB	0.5	(0-1)	Uniform	Model Assumption
β_S^0	TB transmission coefficient until t_s	6.5	(6.0-7.0)	Uniform	Calibrated to Target Incidence
d_S	Rate of decline in TB transmission coefficient, starting in 2000	-0.0125	(-0.025 - 0)	Uniform	Calibrated to Target Incidence

†Sampling ranges represent the 2.5th to 97.5th percentiles of unbounded distributions and lower to upper bounds of uniform distributions.
*Protection by latent infection is assumed equal between infections with DS-TB and MDR-TB and assumed to be unassociated with differences in the transmission efficiency of the two strains.

Table S2: HIV and TB/HIV Parameter Prior Distributions

	Description	Median	Sampling Range	Distribution	References
μ^H	Added mortality rate of HIV+, High CD4 populations (per year)	0.05	(0.03-0.07)	Lognormal	[25]
μ^L	Added mortality rate of HIV+, Low CD4 populations (per year)	0.27	(0.23-0.33)	Lognormal	[25]
μ^A	Added mortality rate of HIV+, receiving ART populations (per year)	0.01	(0.008-0.013)	Lognormal	[26]
$\mu_{tb}{}^L$	Excess added mortality rate of HIV+, Low CD4 individuals infected with untreated active TB (per year)	0.8	(0.5-1.3)	Lognormal	[27]
φ_H	Rate of transition from HIV+, High CD4 to HIV+, Low CD4 (per year)	0.2	(0.15-0.26)	Lognormal	[28,29]
k_H	Reduced probability of initiating ART for HIV+, High CD4 populations relative HIV+, Low CD4 populations (after 2006 only)	0.33	(0.11-1.0)	Logit-normal	[30]
ρ^H	Relative increase in the probability of rapid progression of TB in HIV+, High CD4 populations	2.9	(1.8-4.7)	Lognormal	[27]
ρ^L	Relative increase in the probability of rapid progression of TB in HIV+, Low CD4 populations	8	(6.3-10.1)	Lognormal	[27]
ρ^A	Relative increase in the probability of rapid progression of TB in HIV+, on ART populations	2.9	(1.8-4.7)	Lognormal	[27]
λ^H	Relative reduction in the probability of protection against rapid progression upon reinfection in HIV+, High CD4 populations	0.25	(0-0.5)	Uniform	[31]
λ^L	Relative reduction in the probability of protection against rapid progression upon reinfection in HIV+, Low CD4 populations	0.25	(0-0.5)	Uniform	[31]
λ^A	Relative reduction in the probability of protection against rapid progression upon reinfection in HIV+, on ART populations	0.25	(0-0.5)	Uniform	[31]
r^H	Relative increase in the rate of reactivation in HIV+, High CD4 populations	34	(20-60)	Lognormal	[32,33]
r^L	Relative increase in the rate of reactivation in HIV+, Low CD4 populations	67	(31-158)	Lognormal	[32,33]
r^A	Relative increase in the rate of reactivation in HIV+, on ART populations	34	(20-60)	Lognormal	[32,33]
ν^H	Relative reduction in the rate of spontaneous resolution in HIV+, High CD4 populations	0.5	(0-1)	Uniform	Model Assumption
ν^L	Relative reduction in the rate of spontaneous resolution in HIV+, Low CD4 populations	0.25	(0-0.5)	Uniform	Model Assumption
ν^A	Relative reduction in the rate of spontaneous resolution in HIV+, on ART populations	0.5	(0-1)	Uniform	Model Assumption
i^H	Relative reduction in the infectiousness of HIV+, High CD4 populations	0.89	(0.53-0.98)	Logit-normal	[34,35]
i^L	Relative reduction in the infectiousness of HIV+, Low CD4 populations	0.5	(0.12-0.88)	Logit-normal	[34]
i^A	Relative reduction in the infectiousness of HIV+, on ART populations	0.89	(0.53-0.98)	Logit-normal	[34,35]
k_{tb}	Relative increase in the rate of ART initiation in HIV+ TB patients receiving any form of TB treatment	2.4	(1.0-5.6)	Lognormal	[36]
x^A	Relative reduction in the duration before TB treatment initiation in HIV+ TB patients receiving ART	0.43	(0.3-0.6)	Lognormal	[37]

†Sampling ranges represent the 2.5th to 97.5th percentiles of unbounded distributions and lower to upper bounds of uniform distributions.

Table S3: MDR-TB Parameter Prior Distributions

	Description	Median	Sampling Range	Distribution	References
t_R^0	Time since emergence of modern MDR-TB strains (years)	30	(20-45)	Lognormal	[38,39]
α^0	Risk of acquired multidrug-resistance during first-line therapy, new DS-TB patients	0.004	(0.0015-0.01)	Logit-normal	[22]
α^P	Increase in risk of acquired multidrug resistance, retreatment DS-TB patients (multiplicative factor)	2	(1-3)	Uniform	[20,40]
b	Pre-treatment loss to follow up after MDR-TB diagnosis	0.05	(0.025-0.10)	Logit-normal	[3,16]
σ_2	MDR-TB treatment success (fraction of MDR-TB patients who successfully complete treatment if adherent; includes those who may relapse)	0.77	(0.66-0.85)	Logit-normal	[41–43]
i_2	Infectiousness during the first six months of effective MDR-TB therapy, relative to untreated active TB	0.1	(0-0.2)	Uniform	[42,44*]
δ_1	Probability of loss to follow up during MDR-TB therapy	0.11	(0.04-0.25)	Logit-normal	[41,45]
ω_2	Relapse risk after successful completion of conventional MDR-TB treatment	0.04	(0.015-0.1)	Logit-normal	[42,46]
E_R	Transmissibility of drug-resistant strain, relative to drug-susceptible strain at the time of MDR emergence	0.6	(0.38-0.94)	Lognormal	[47–49]
d_R	Annual rate of increase in MDR-TB Transmission coefficient (in the Shrinking Deficit scenario)	0.0075	(0-0.015)	Uniform	Model Assumption
t_R	Year of start of increase in MDR-TB Transmission coefficient (as a percentile of [t_R^0, 2016]	0.5	(0-1)	Uniform	Model Assumption

†Sampling ranges represent the 2.5th to 97.5th percentiles of unbounded distributions and lower to upper bounds of uniform distributions.

*Evidence from Ref. 44 indicate that patients receiving effective second-line therapy are much less infectious than those receiving ineffective therapy; we therefore set a floor of 0%. Evidence from Ref. 42 indicate that 10% of MDR-TB patients remain culture-positive by 6 months of treatment and nearly half remain culture-positive at 2 months (though likely with reduced infectiousness); we therefore set a ceiling of 20%.

Table S4: Calibration Targets

	Total TB Incidence[a,b] (per 100,000)		HIV-Infected Incident TB[b,c] (%)		MDR-TB[d] in New Cases (%)		MDR-TB[d] in Previously-Treated Cases (%)	
	Year	Median (Range)	Year	Median (Range)	Year	Mean (Range)	Year	Mean (Range)
**South Africa**	2005	932 (603-1331)	2012	63.2 (62.2-64.2)	2002	1.6 (1.1-2.1)	2002	6.6 (4.9-8.2)
	2010	948 (710-1219)			2013	2.1 (1.5-2.7)	2013	4.6 (3.2-6.0)
	2015	834 (539-1190)						
**Vietnam**	2005	176 (114-252)	2015	4.3 (4.0-4.6)	1996	2.3 (1.3-3.8)	1996	18.2 (11.9-24.5)
	2010	155 (114-202)			2002	2.3 (1.1-3.6)	2002	19.3 (14.0-25.0)
	2015	137 (110-166)			2005	2.7 (2.0-3.7)	2005	23.3 (16.7-29.9)
					2011	4.0 (2.5-5.4)	2011	18.2 (11.9-24.5)

[a]Incidence estimates were taken from WHO country reports (as published with the 2016 Global TB Report) [3].
[b]These estimates were modeled as independent beta distributions with bounds defined by the estimated 95% confidence intervals.
[c]HIV targets represent the proportion of HIV-infected populations among all incident TB cases. Estimates were taken from the 2012 TB drug resistance survey in South Africa [4] and the 2016 WHO estimate in Vietnam. [d]MDR-TB targets represent the proportion of MDR-TB cases among all recent TB diagnoses (defined as any population transitioning from a state of active, untreated TB to a state of TB diagnosis/treatment). Estimates were taken from national drug resistance surveys in South Africa [4,50] and Vietnam [51]. These estimates were modeled as independent normal distributions with two standard deviations set equal to half the widths of the estimated 95% confidence intervals

Bayesian Model Comparison

To compare the performance of our model scenarios, we calculated a Bayes Factor for each pair of scenarios in each country. A Bayes Factor is traditionally defined as the posterior odds to prior odds ratio [52]. For posterior distributions from any two models $p(\theta_1|y)$ and $p(\theta_2|y)$ and data y (our empirical calibration targets), a Bayes Factor can be defined as:

$$BF = \frac{p(\theta_1|y)/p(\theta_1)}{p(\theta_2|y)/p(\theta_2)} \quad (46)$$

(In this notation, $p(\theta_1)$ and $p(\theta_2)$ represent the prior distributions of each model.) As with common Monte Carlo approximations to Bayesian inference, if independent samples are drawn from $p(\theta_1)$ and $p(\theta_2)$, the BF may be consistently estimated by [52]:

$$\frac{n_2 \sum_{j=1}^{n_1} L(\theta_{j_1}; y)}{n_1 \sum_{j=1}^{n_2} L(\theta_{j_2}; y)} \qquad (47)$$

In our approach, the number of independent simulations run in each scenario's prior distribution was equal ($n_1 = n_2$). Therefore, the BF is approximated by the ratio of the sums of likelihood values for the *n* prior simulations (before resampling) in each scenario ($\sum_{j=1}^{n} L\,(\theta_j; y)$). When presented in the text, the Constant Deficit scenario is used as the denominator model $p(\theta_2|y)$ in calculating a BF unless noted otherwise.

Replication of Previous Findings

After review of our primary results, we noted important discrepancies in our projections of the MDR-TB epidemic in South Africa under the No Deficit scenario and those published in a similar study by Sharma and colleagues in which the transmission efficiency of DS-TB and MDR-TB were assumed to be identical [53]. Notably, their work projected the relative incidence of MDR-TB in South Africa to be 5.7% (95% UR: 3.0-7.6%) by 2040, significantly lower and with less variance than our estimates. In an attempt to reconcile these projections, we undertook to identify the source(s) of these discrepancies in our approach.

In reviewing the methodology of the previously published study, we identified several parameters with comparable surrogates in our model where our values diverged. Whereas our colleagues parameterized the 5-year probability of latent TB reactivation with a median of 2.9%, we parameterized the rate of TB reactivation r^0 with a median of 0.1 per 100 person-years (equivalent to a 5-year probability of 0.5%). Additionally, they parameterized the median

probabilities of acquiring active TB and latent TB from an infectious index case as 3.3% and 51%, respectively. It follows that, among those who acquire a new TB infection, approximately 6% would bypass the latent state (3.3/(51+3.3). We parameterized the comparable median probability of rapid progression upon acquiring a new TB infection ρ^0 as 14%. Finally, our colleagues' approach to the rate of treatment initiation used a case detection rate of 75% with a wide prior distribution (3-98%) for the probability that TB patients will initiate effective treatment following detection. The posterior of this distribution is significantly narrower (6-26%) with a low median value (11.3%). This implies that only 8% of DS-TB patients would initiate effective TB treatment in South Africa; equivalently, the mean time between TB onset and treatment initiation would be 11 years. By comparison, we parameterized the mean delay between TB onset and treatment initiation as 12 months.

To replicate these previously published results, we altered our parameter priors in a stepwise manner. First, in our Slower Epidemic scenario, we decreased the prior probability of rapid progression ρ^0 upon new infection from 14% (95% range: 7-25%) to 4% (95% range: 1-15%) and increased the range for the rate of latent TB reactivation r^0 from 0.05-0.2 per 100 person-years to 0.01-1.0 per 100 person-years and executed our No Deficit scenario as before. Afterwards, in our Delayed Treatment scenario, we additionally increased the prior duration of time between TB onset and treatment initiation from 12 months (95% range: 8-18 months) to 10 years (95% range: 7.5-12.5 years). To assess model performance, we calculated Bayes Factors comparing these two new scenarios with our No Deficit scenario and our Constant Deficit scenario.

Sensitivity Analyses

We implemented two strategies in our sensitivity analyses. For each parameter in each scenario, we calculated the partial rank correlation coefficient (PRCC) between the parameter's posterior values and two primary outcomes of interest generated from simulations with those values: the absolute 2040 MDR-TB incidence and the fold change in the relative MDR-TB incidence (as a proportion of all TB) between 2016 and 2040. This multivariate analysis evaluates the strength of rank correlation between a parameter input value and the associated outcome value, adjusting for all other parameters. Additionally, in a univariate approach, we stratified outcome values across quintiles of parameter values. Briefly, for each parameter in each scenario, posterior simulations were ordered by parameter value (least to greatest). The bottom 20% of simulations (those with the lowest parameter values) and the top 20% of simulations (those with the highest parameter values) were identified and outcomes of simulations in each quintile were plotted. In cases where a parameter is highly influential on an outcome, a clear separation is expected between the distributions of the outcome in the lowest and highest quintiles of the parameter's value. In cases where a parameter is not influential, the distribution of outcomes is not expected to differ significantly between quintiles of the parameter's value. Additionally, we compared the prior distribution of sampled values with the posterior distribution each input parameter after calibration to illustrate values for specific parameters that were inconsistent with calibration targets.

SUPPLEMENTARY RESULTS

Calibration

As described in the Methods, our scenarios were calibrated in a two-stage semi-Bayesian algorithm. The results of the first stage of calibration – using estimates of absolute TB incidence and HIV among incident TB cases – are presented in Fig. S4. The results of the second stage of calibration – using empirical estimates of MDR-TB incidence – are presented in the Results (Fig. 2 and Fig. 3). All Bayes Factors calculated between the three primary scenarios examined are presented in Table S5.

Figure S4: TB and HIV Incidence Calibration Results

A – South Africa

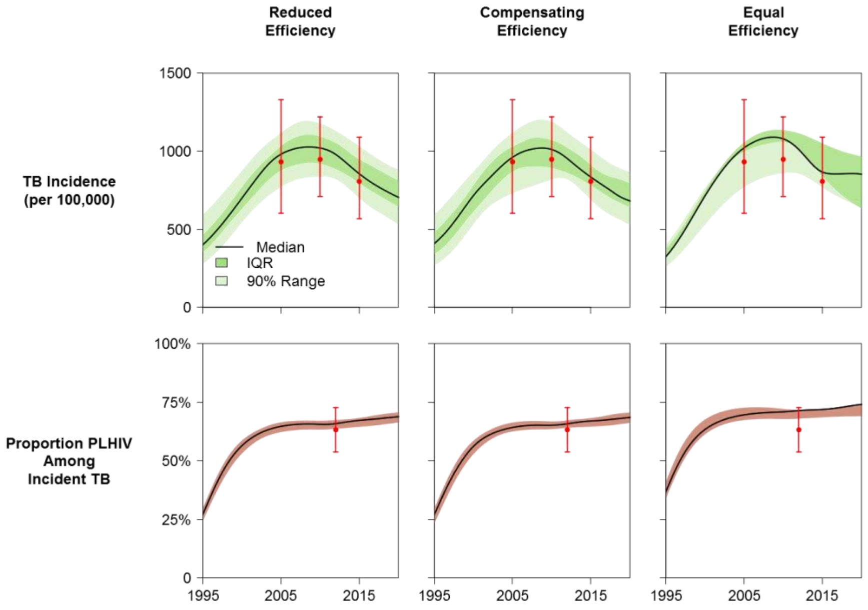

B – Vietnam

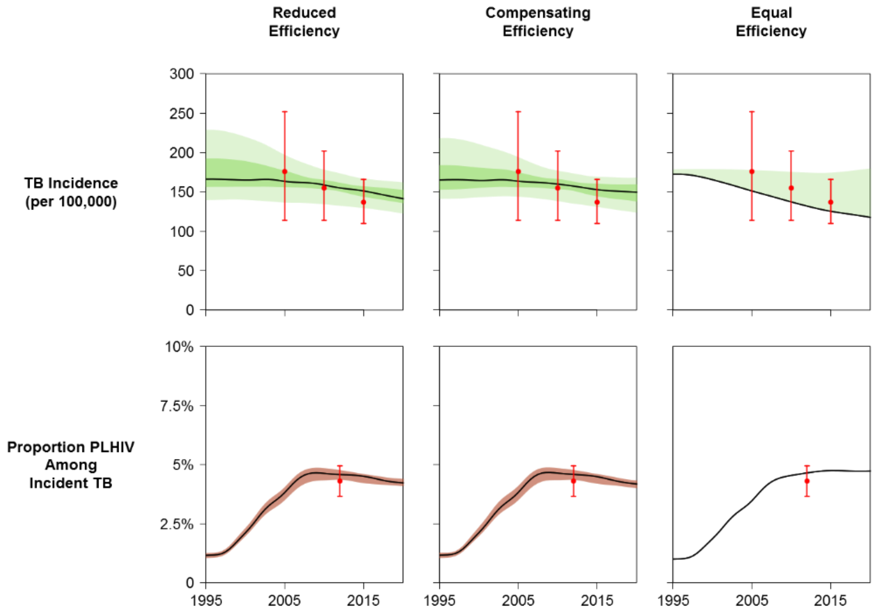

Simulated epidemics are weighted according to how well each reproduced empirical calibration targets (historical estimates of absolute TB incidence and the proportion of incident TB cases infected with HIV). Red points represent median values and bounds for calibration targets drawn from WHO estimates and national survey data. IQR represents 25th to 75th percentiles and the 90% range represents the 5th to 95th percentiles of posterior simulations.

Table S5: Model Comparison with Bayes Factors

BF Numerator Scenario[a]	BF Denominator Scenario[b]	South Africa BF[c]	Vietnam BF[d]
Constant Efficiency Deficit	No Efficiency Deficit	67,380,983	3354
Constant Efficiency Deficit	Shrinking Efficiency Deficit	2.6	2.1
Shrinking Efficiency Deficit	No Efficiency Deficit	25,868,566	1607

[a]The model used in the numerator of the calculation of the Bayes Factor (i.e., $p(\theta_1|y)$ in Equation 47 above).
[b]The model used in the denominator of the calculation of the BF (i.e., $p(\theta_2|y)$ in Equation 47 above).
[c]The BF corresponding to the performance of the numerator model relative to the denominator model in South Africa.
[d]The BF corresponding to the performance of the numerator model relative to the denominator model in Vietnam

Model Projections

The primary outcomes of interest in our study were the projected changes in absolute and relative incidences of MDR-TB over time. Projections of the absolute MDR-TB incidence in the Constant Deficit and Shrinking Deficit scenarios are presented in the Results (Fig. 4). For these scenarios, we present projections of the relative incidence of MDR-TB (as a proportion of all TB) in Fig. S5. In the case of the No Deficit scenario, we present projections of the absolute and relative MDR-TB incidence in Fig. S6. Additionally, we investigated several secondary outcomes of interest in all scenarios. Projections of the absolute TB incidence (used to calculate the relative MDR-TB incidence presented in the Results and Fig. S5) are illustrated in Fig. S7. The proportion of incident MDR acquired during recent first-line therapy is presented in Fig. S8.

Figure S5: Projections of the Relative Incidence of MDR-TB

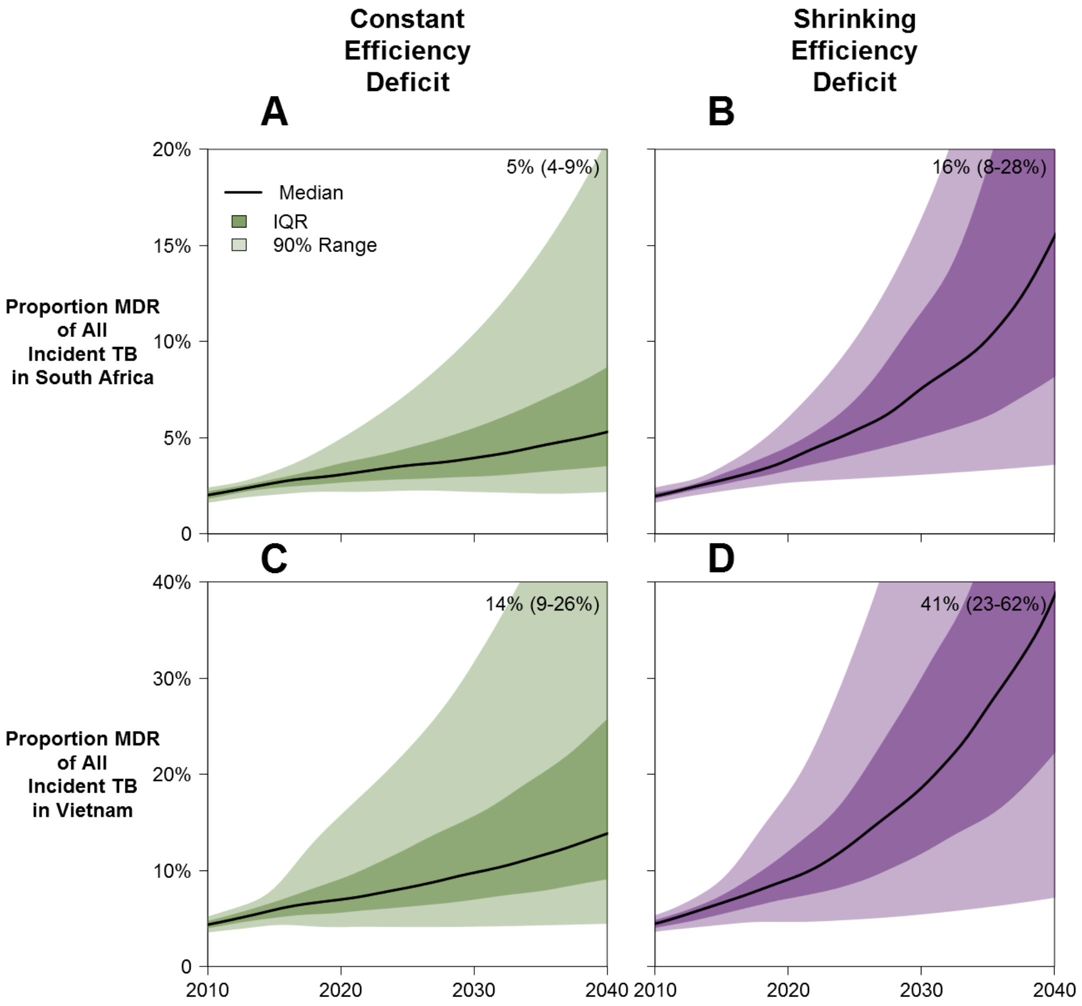

Simulated MDR-TB epidemics in South Africa and Vietnam were projected from 2010 to 2040. Panels A and B illustrate the projections of each scenario in South Africa, while panels C and D illustrate the projections of each scenario in Vietnam. The 2040 projected median (IQR) values are included in the upper right of each panel. IQR represents 25th to 75th percentiles and the 90% range represents the 5th to 95th percentiles of posterior simulations.

Figure S6: Projections of the MDR-TB Burden in the No Deficit Scenario

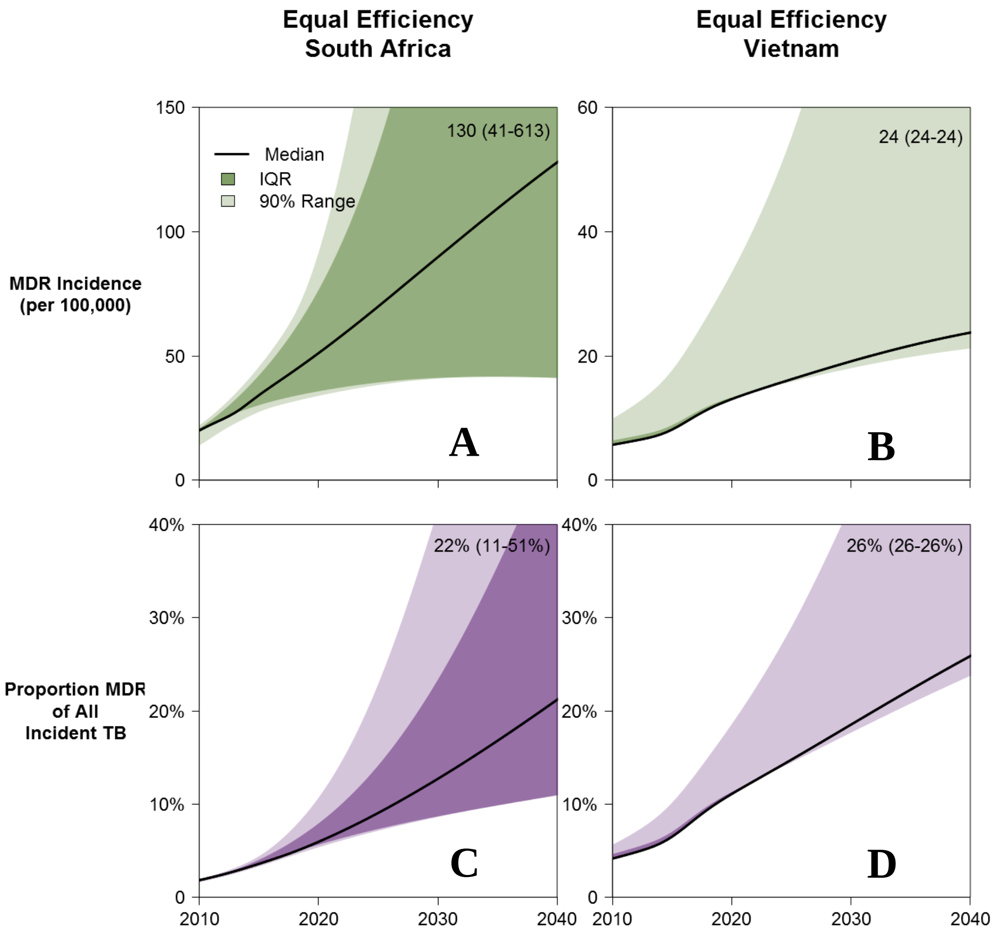

Simulated MDR-TB epidemics were projected from 2010 to 2040. Panels A and B demonstrate the projected absolute MDR-TB incidence in each country, while Panels C and D demonstrate MDR-TB as a proportion of all incident TB. The 2040 projected median (IQR) values are included in the upper right of each panel. IQR represents 25th to 75th percentiles and the 90% range represents the 5th to 95th percentiles of posterior simulations.

Figure S7: Projections of the Absolute Burden of TB

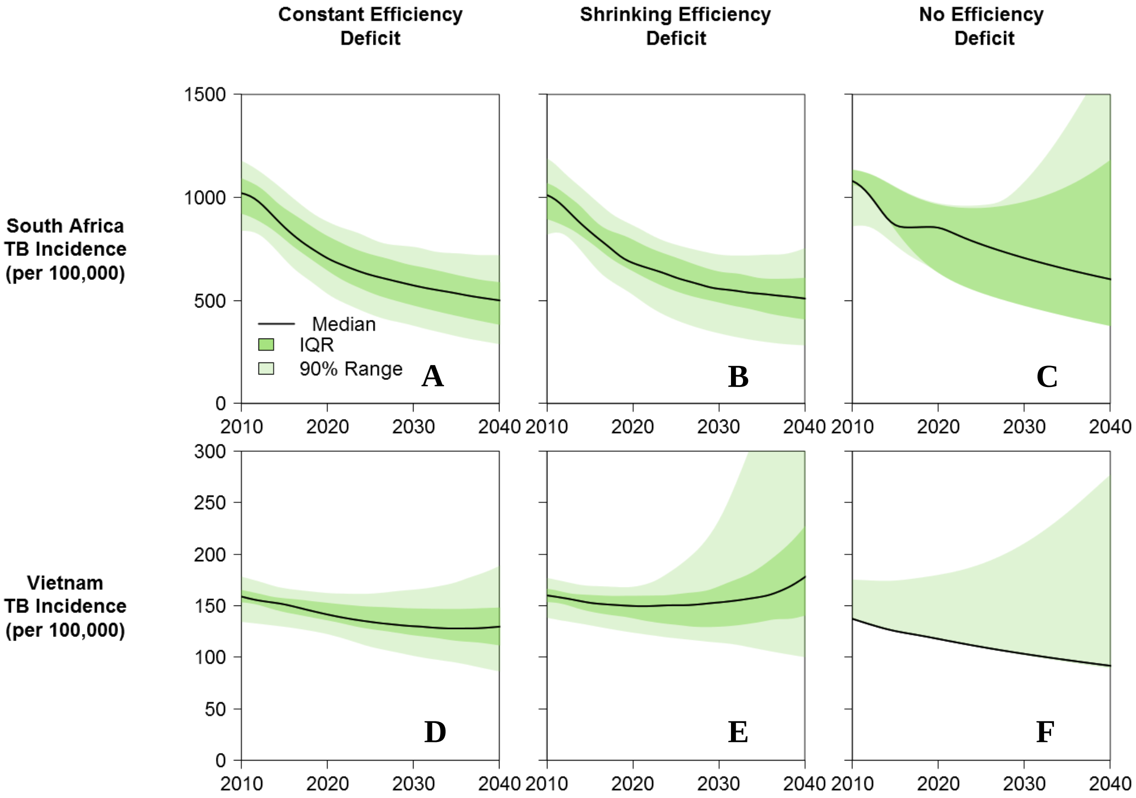

Simulated MDR-TB epidemics were projected from 2010 to 2040, and the absolute incidence of all forms of TB (DS-TB and MDR-TB) are illustrated. Panels A and D represent projections in the Constant Deficit scenario in each country; panels B and E represent projections in the Shrinking Deficit scenario; panels C and F represent projections in the "Equal Transmission Efficency" scenario. The 2040 projected median (IQR) values are included in the upper right of each panel. IQR represents 25th to 75th percentiles and the 90% range represents the 5th to 95th percentiles of posterior simulations.

Figure S8: Projections of Acquired Drug Resistance

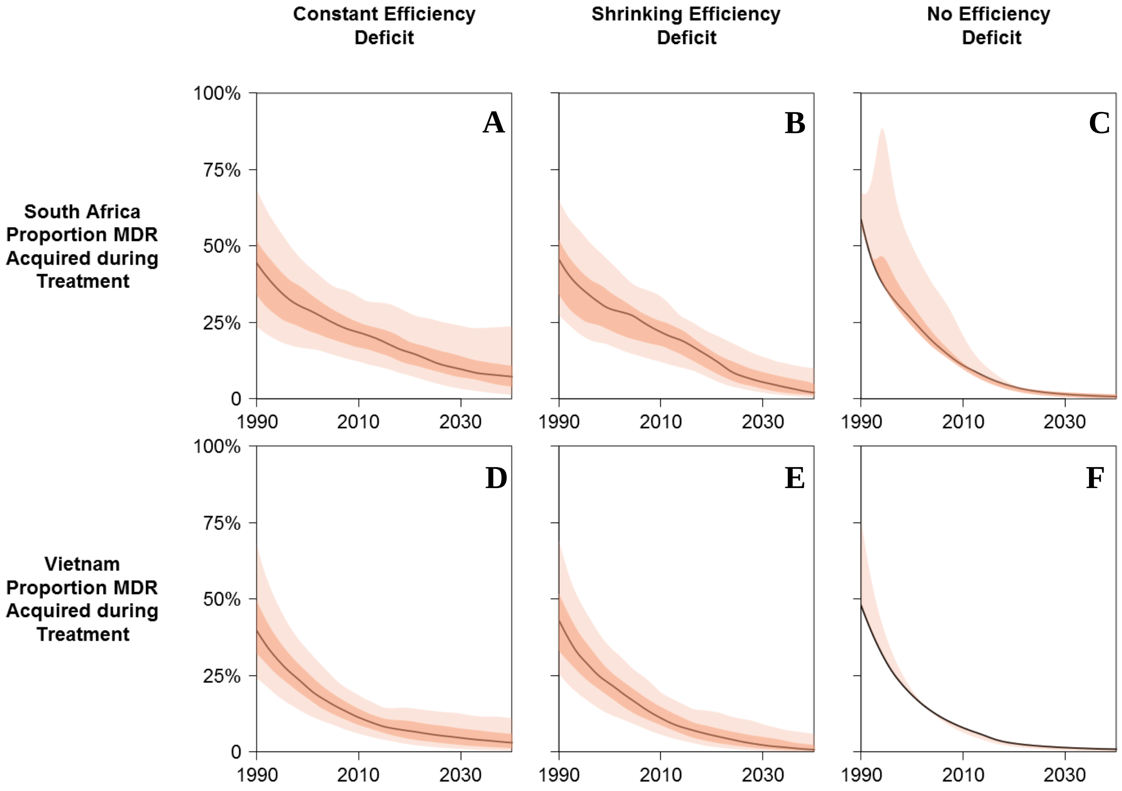

In our model, the acquisition of drug resistance (ADR) is defined as occurring at the time any population exits a DS-TB treatment state (B_{1eS} or B_{1iS}) and enters a state of symptomatic MDR-TB, will relapse with symptomatic MDR-TB, or initiates MDR-TB treatment (A_{PR} , B_{1iPR}, W_R, B_{2e1}, B_{2i}, or C_R). The transmission of drug resistance (in the calculation of proportion ADR) is defined as occurring at the time any asymptomatic, treatment-naïve population experiences the first onset of detectable MDR-TB (from S or L_R to E_R). The projected proportion of all recent-onset MDR-TB due to ADR is illustrated above. Panels A and D represent projections in the Constant Deficit scenario in each country; panels B and E represent projections in the Shrinking Deficit scenario; panels C and F represent projections in the No Deficit scenario. The 2040 projected median (IQR) values are included in the upper right of each panel. IQR represents 25th to 75th percentiles and the 90% range represents the 5th to 95th percentiles of posterior simulations.

Replication of Previous Findings

In our attempt to replicate the findings of Sharma and colleagues [53] using our No Deficit scenario, we first altered prior distributions for two parameters – the probability of rapid progression upon initial TB infection and the rate of reactivation upon latent infection – such that simulated TB epidemics were driven less by rapidly progressing TB and driven more by the reactivation of latent TB, leading to a more slowly developing TB epidemic. A number of epidemiological studies have examined primary progressive TB and TB reactivation, with some variation in estimates between studies and between demographic groups within studies [7–11]. These estimates may be parameterized in several ways, and the modified values that adopted for this Slower Epidemic scenario may be consistent with this body of literature.

The calibration results of this Slower Epidemic scenario are presented in Fig. S9 in comparison with the results of our Constant Deficit and No Deficit scenarios. This Slower Epidemic scenario was supported even less by empirical data relative to our No Deficit scenario (BF=0.01). As the Slower Epidemic becomes driven by reactivation, a high proportion of incident TB cases are among those living with HIV; this higher than expected proportion of HIV in TB cases performs poorly to our calibration of HIV/TB coinfection targets. In the Slower Epidemic scenario, we project MDR-TB will account for 17% of all incident TB by 2040 (see Fig. S10).

Figure S9: Calibration Results of a Slower Epidemic Scenario in South Africa

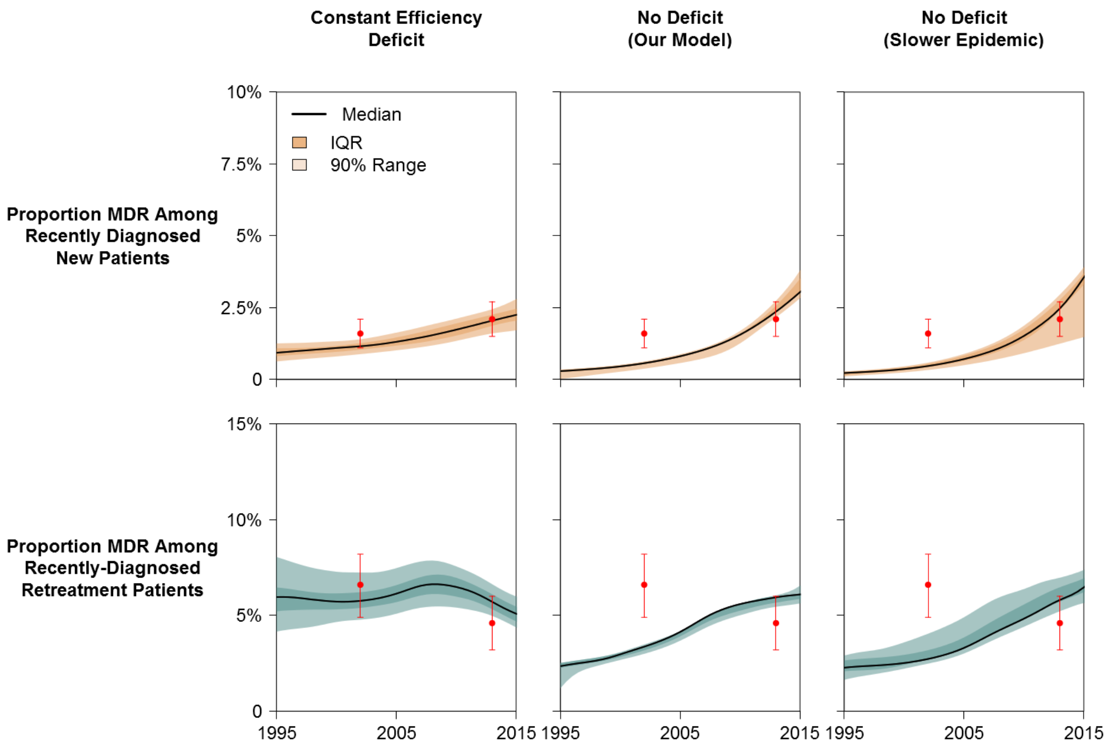

Simulated epidemics are weighted according to how well each reproduced empirical calibration targets. Red points represent median and 95% confidence intervals for calibration targets drawn from national survey data. For comparison, results from the Constant Deficit scenario and the No Deficit scenario (Fig. 2) are reproduced here. IQR represents 25th to 75th percentiles and the 90% range represents the 5th to 95th percentiles of posterior simulations.

Figure S10: Projections of MDR-TB Burden in a Slower Epidemic

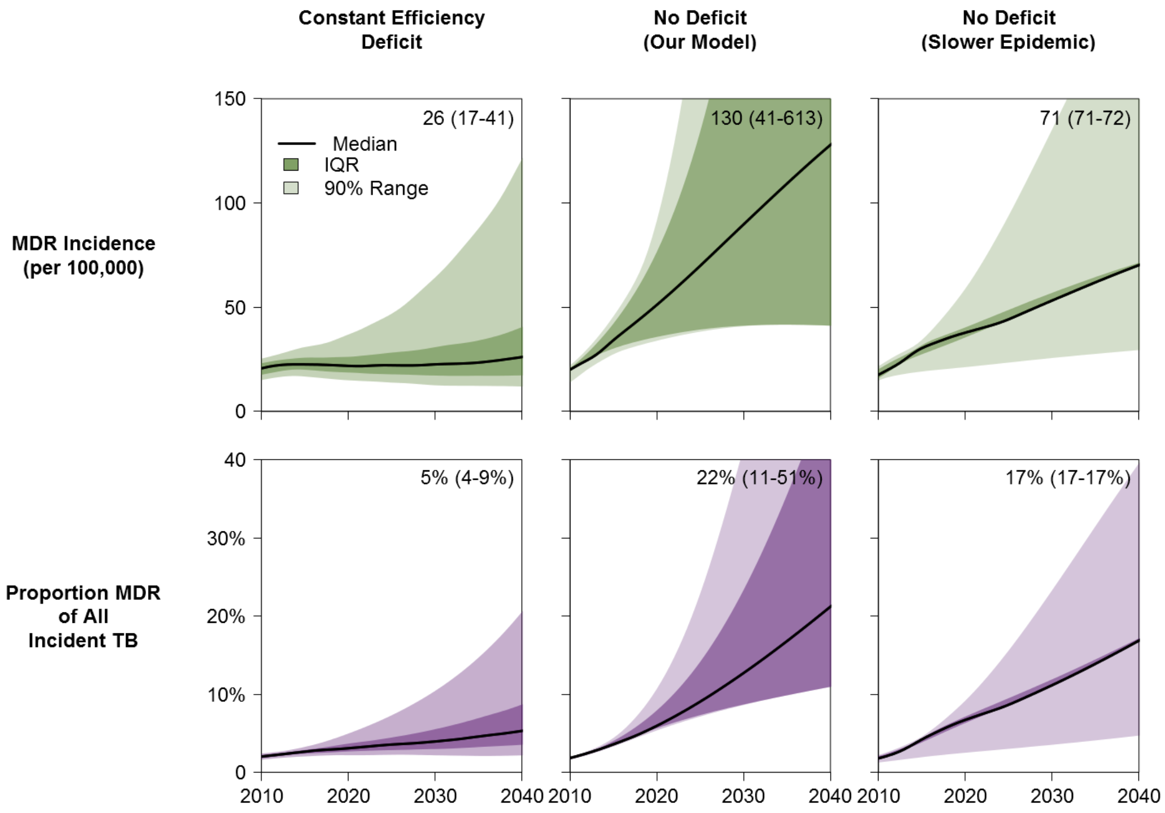

Simulated MDR-TB epidemics in South Africa were projected from 2010 to 2040. Panels in the top row illustrate the projected absolute MDR-TB incidence, while panels in the lower row illustrate MDR-TB as a proportion of all incident TB. For comparison, results from the Constant Deficit scenario (Fig. 3) and the No Deficit scenario (Fig. S7 above) are reproduced here. The 2040 projected median (IQR) values are included in the upper right of each panel. IQR represents 25th to 75th percentiles and the 90% range represents the 5th to 95th percentiles of posterior simulations.

These results were still meaningfully higher than the estimates published by Sharma and colleagues [53]. We therefore adjusted this scenario further by increasing the prior distribution of the duration of delay between the onset of TB and the initiation of care from a median of 12 months to a median of 10 years, comparable to the posterior probability of effective DS-TB treatment initiation published by Sharma and colleagues. In this Delayed Treatment scenario, few TB patients (DS-TB or MDR-TB) receive treatment of any kind, having died of TB or self-cured before treatment begins. As a result, MDR-TB exerts little competitive advantage over DS-TB (through lower probabilities of cure and longer durations of symptomatic, infectious disease). With this additional adjustment, the Delayed Treatment scenario was better supported by empirical data than the Slower Epidemic scenario (BF>10^8) but still more poorly supported relative to our Constant Deficit scenario (BF=0.08) and our Shrinking Deficit scenario (BF=0.21) (for calibration results and projections of the Delayed Treatment scenario, see Results Fig. 6)

Sensitivity Analyses

As described in the Supplementary Methods, we performed multivariate sensitivity analyses using PRCCs and univariate sensitivity analyses using parameter quintiles associated with two primary outcomes: the absolute incidence of MDR-TB in 2040 and the fold change in the relative incidence of MDR-TB between 2016 and 2040. Univariate analysis of the absolute incidence of MDR-TB in South Africa was presented in Fig. 6, and the univariate analysis of the relative incidence of MDR-TB in South Africa is presented in Fig. S11 below. Univariate analysis of the absolute incidence of MDR-TB in Vietnam is presented in Fig. S12. Multivariate analysis of the absolute incidence of MDR-TB in both countries is presented in Fig. S13 below. Posterior distributions of parameter values resampled during calibration of the South Africa MDR-TB epidemic in the Constant Deficit and Shrinking Deficit scenarios are compared with the prior distributions used for Latin hypercube sampling in Figs. S14-16.

Figure S11: Univariate Sensitivity Analysis – Relative MDR-TB Incidence in South Africa

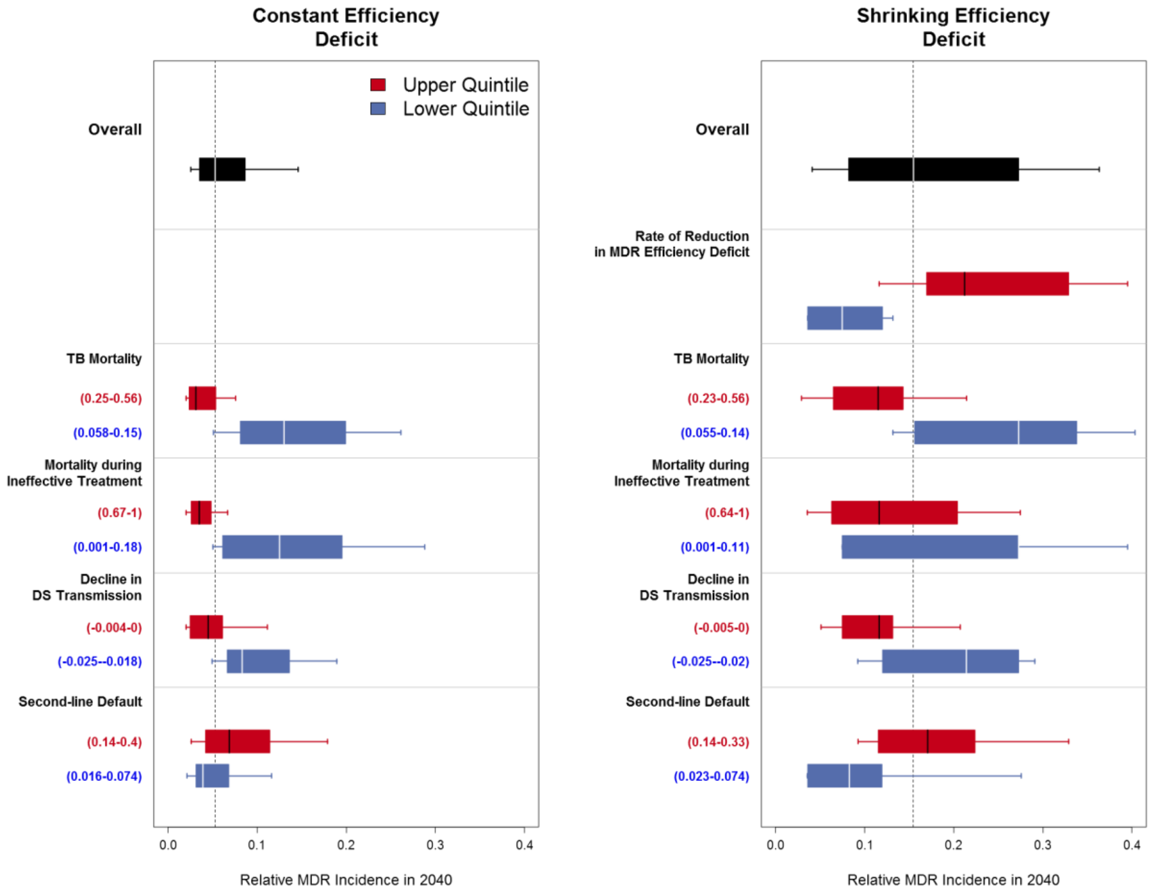

The top 5 parameters which most strongly impact the distributions of relative MDR-TB incidence in 2040 are displayed. Each boxplot represents the distribution of values for the primary outcome (the incidence of MDR-TB in 2040) within a given set of simulations. Pairs of boxplots represent groups of simulations categorized by values of a single input parameter: red boxplots represent the outcomes of those simulations with parameter values in the upper 20% of all simulations; blue boxplots represent the outcomes of those simulations with parameter values in the lower 20% of all simulations. More influential parameters demonstrate a greater separation of the distributions of outcome between simulations in the upper quintile and simulations in the lower quintile of parameter values. To the left of each panel are included the input parameter values corresponding to the accompanying quintile. In black is represented the overall distribution of the outcome across all simulations and the median estimate is drawn as a vertical dotted line. Boxes represent the median, 25th, and 75th percentiles of the distribution of outcomes; whiskers

represent the 5th and 95th percentiles of the distribution of outcomes. In the Constant Deficit model, parameters involving the reduction in MDR-TB transmission efficiency deficit are excluded.

Figure S12: Univariate Sensitivity Analysis – Absolute MDR-TB Incidence in Vietnam

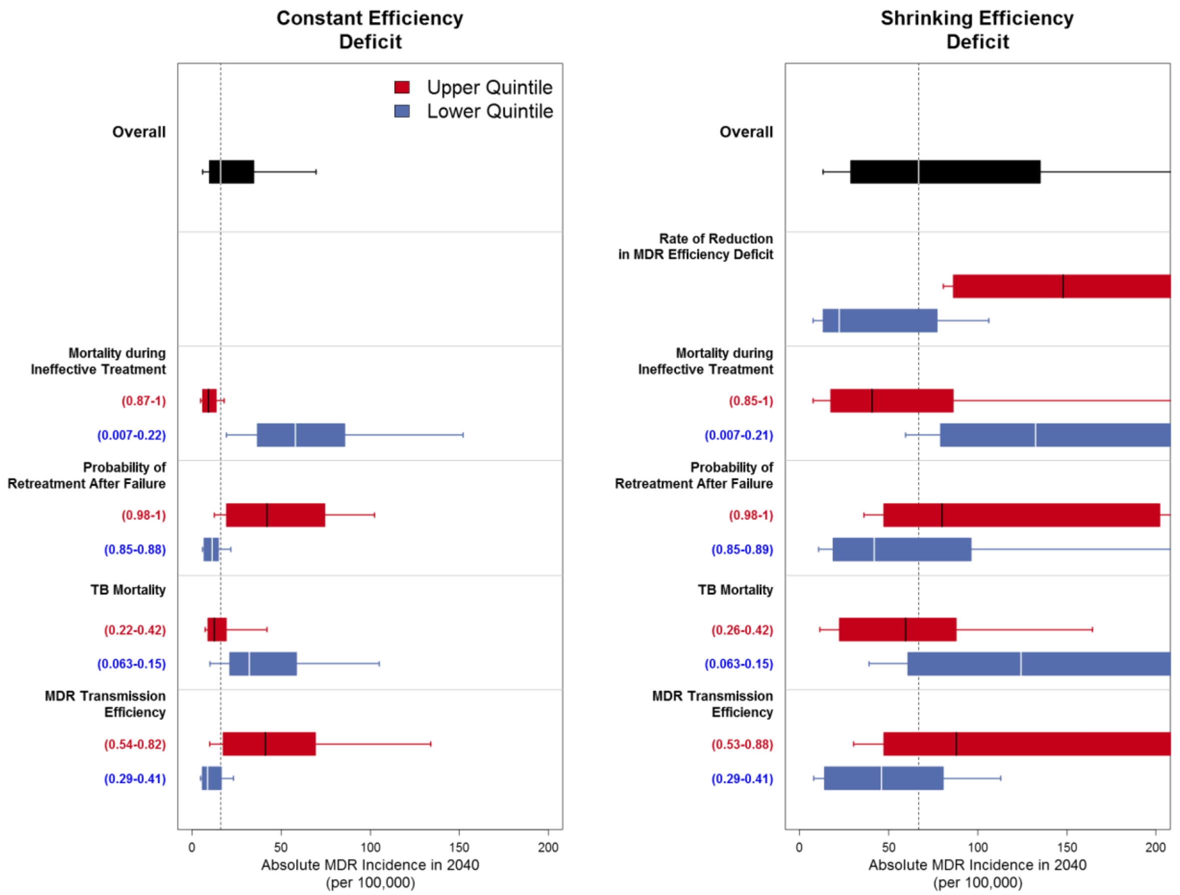

Figure S13: Multivariate Sensitivity Analysis – Absolute MDR-TB Incidence

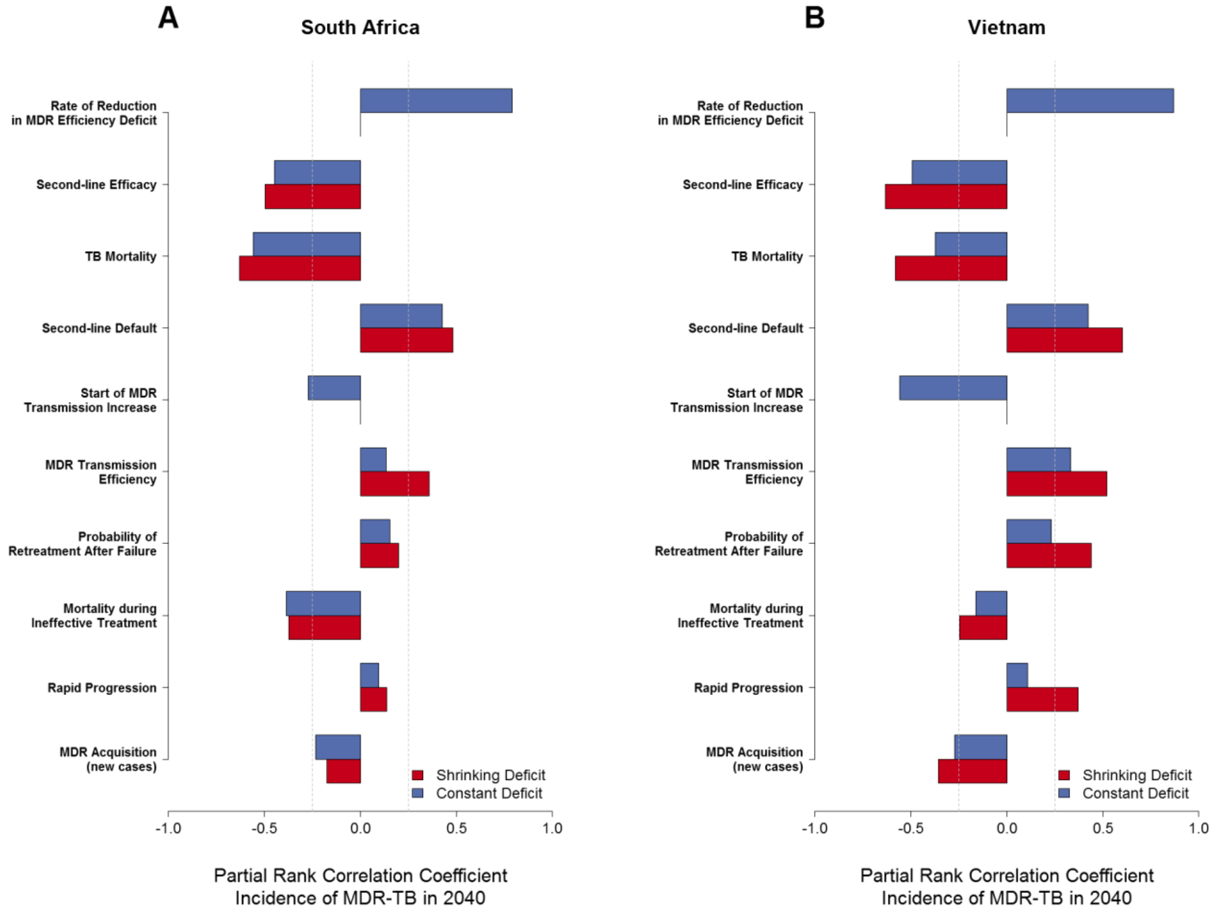

Plots represent the top 10 parameters of strongest correlation (positive or negative) with the incidence of MDR-TB in 2040. Each bar represents the partial rank correlation coefficient of the association between each model parameter and the outcome, adjusting for all other parameters in the model. Panel A presents the results from South Africa, while panel B presents the results from Vietnam.

Figure S14: TB Natural History Parameter Distributions – South Africa

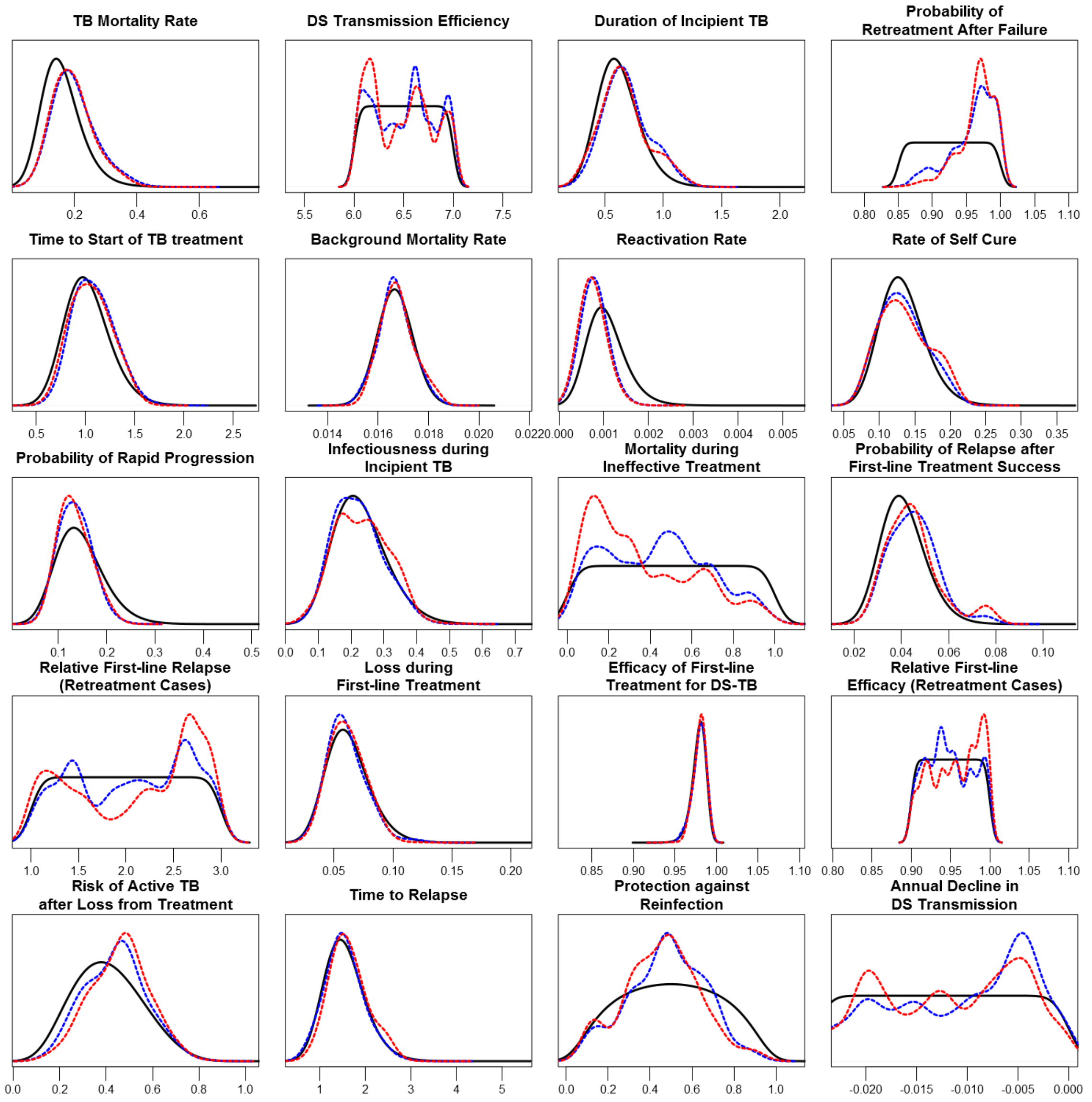

The probability distribution function used to sample initial values for each parameter (the prior distribution) is illustrated in **Black** in each plot. (Identical prior distributions were used to sample parameter values in both Constant Deficit and Shrinking Deficit scenarios.) The distribution of values for each parameter after resampling in the Constant Deficit scenario is illustrated in **Blue** in each plot. The distribution of values for each parameter after resampling in the Shrinking Deficit scenario is illustrated in **Red** in each plot. See Table S2.1 for full descriptions, sampling ranges, and references for each parameter and prior distribution.

Figure S15: HIV and TB/HIV Parameter Distributions – South Africa

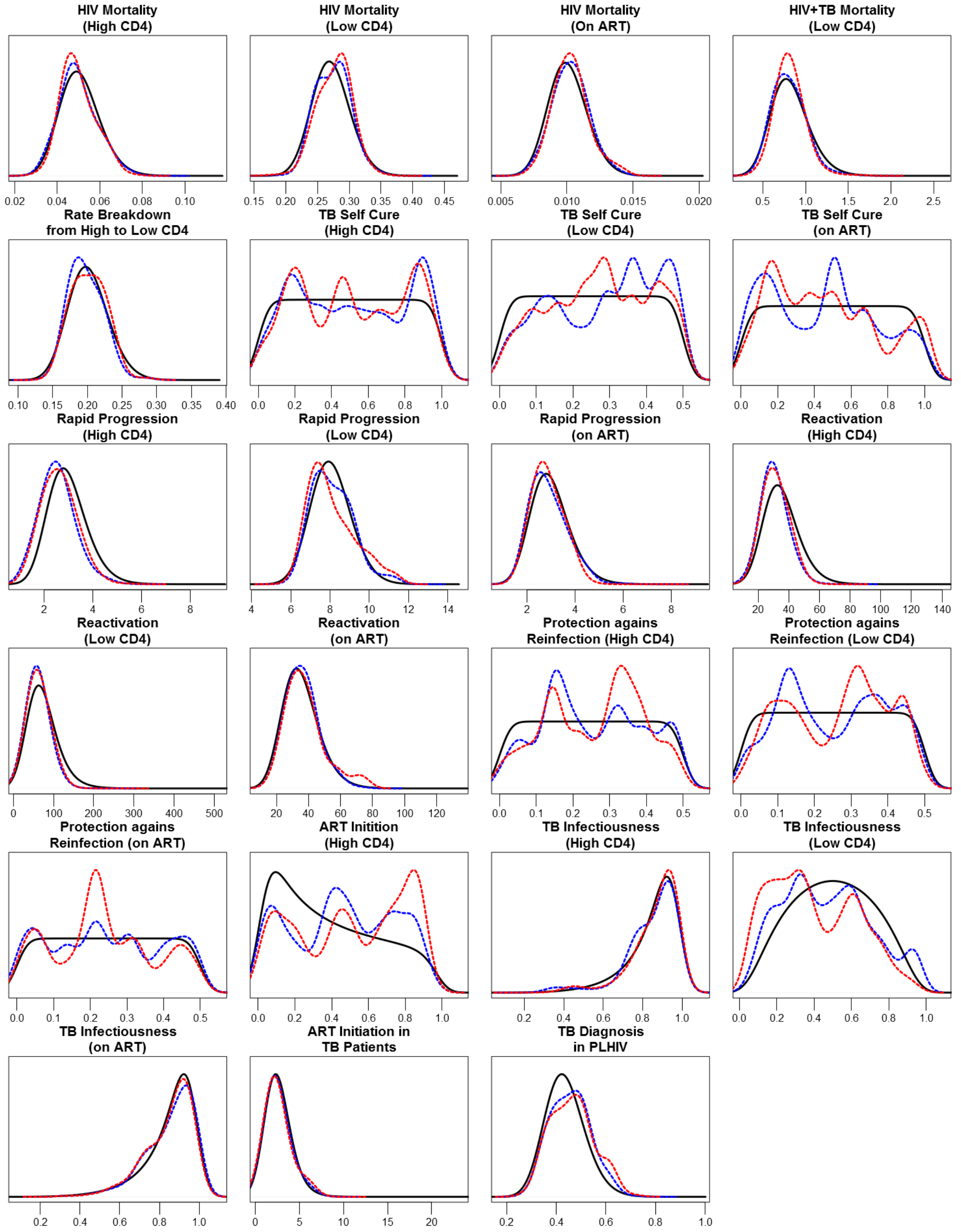

Figure S16: MDR-TB Parameter Distributions – South Africa

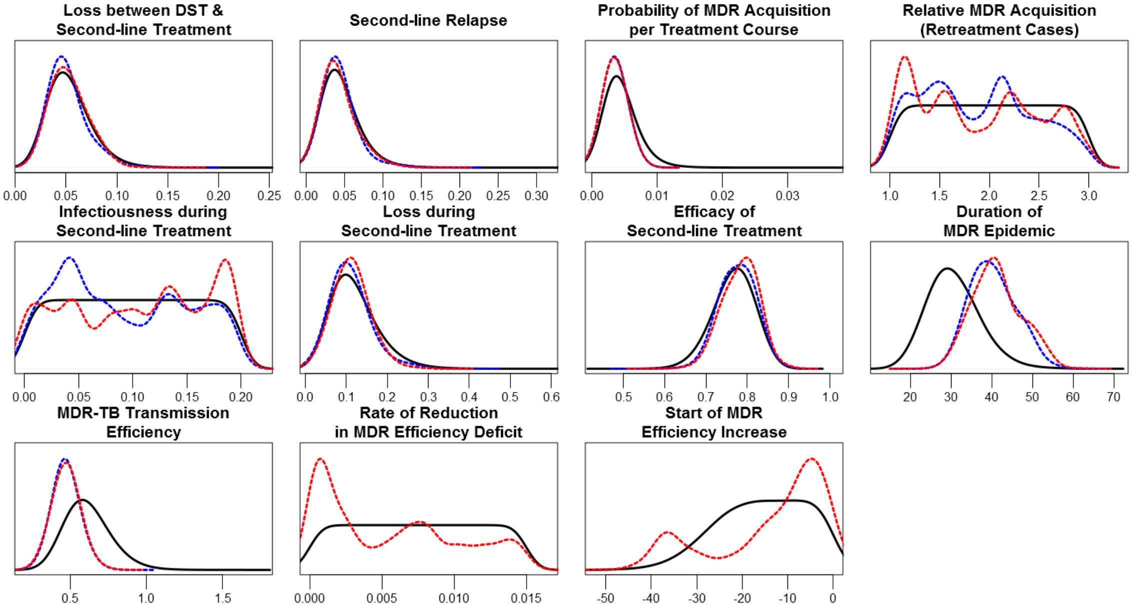

References

1 Smith AFM, Gelfand AE. Bayesian statistics without tears: a sampling-resampling perspective. *Am Stat* 1992; **46**: 84–8.

2 John J, Draper N. An alternative family of transformations. *Appl Stat* 1980; **29**: 190–7.

3 World Health Organization. Global Tuberculosis Report 2017. Geneva, Switzerland: World Health Organization, 2017

4 National Institute for Communicable Diseases (NICD). South African Tuberculosis Drug Resistance Survey 2012–14. 2016

5 World Health Organization (WHO). Global Health Observatory data repository. http://apps.who.int/gho/data/?theme=main&vid=61830. .

6 Tiemersma EW, van der Werf MJ, Borgdorff MW, Williams BG, Nagelkerke NJD. Natural history of tuberculosis: duration and fatality of untreated pulmonary tuberculosis in HIV negative patients: A systematic review. *PLoS One* 2011; **6**: e17601.

7 Vynnycky E, Fine PE. The natural history of tuberculosis: the implications of age-dependent risks of disease and the role of reinfection. *Epidemiol Infect* 1997; **119**: 183–201.

8 Andrews JR, Noubary F, Walensky RP, Cerda R, Losina E, Horsburgh CR. Risk of progression to active tuberculosis following reinfection with Mycobacterium tuberculosis. *Clin Infect Dis* 2012; **54**: 784–91.

9 Horsburgh CR, O'Donnell M, Chamblee S, *et al.* Revisiting rates of reactivation tuberculosis: a population-based approach. *Am J Respir Crit Care Med* 2010; **182**: 420–5.

10 Fox GJ, Barry SE, Britton WJ, Marks GB. Contact investigation for tuberculosis: a systematic review and meta-analysis. *Eur Respir J* 2013; **41**: 140–56.

11 Sloot R, Schim Van Der Loeff MF, Kouw PM, Borgdorff MW. Risk of tuberculosis after recent exposure: a 10-year follow-up study of contacts in Amsterdam. *Am J Respir Crit Care Med* 2014; **190**: 1044–52.

12 Onozaki I, Law I, Sismanidis C, Zignol M, Glaziou P, Floyd K. National tuberculosis prevalence surveys in Asia, 1990-2012: an overview of results and lessons learned. *Trop Med Int Heal* 2015; **20**: 1128–45.

13 Dowdy DW, Basu S, Andrews JR. Is passive diagnosis enough? The impact of subclinical disease on diagnostic strategies for tuberculosis. *Am J Respir Crit Care Med* 2013; **187**: 543–51.

14 Behr MA, Warren SA, Salamon H, *et al.* Transmission of Mycobacterium tuberculosis from patients smear-negative for acid-fast bacilli. *Lancet* 1999; **353**: 444–9.

15 Tostmann A, Kik S V., Kalisvaart NA, *et al.* Tuberculosis Transmission by Patients with Smear-Negative Pulmonary Tuberculosis in a Large Cohort in The Netherlands. *Clin Infect Dis* 2008; **47**: 1135–42.

16 MacPherson P, Houben RM, Glynn JR, Corbett EL, Kranzer K. Pre-treatment loss to follow-up in tuberculosis patients in low- and lower-middle-income countries and high-burden countries: a systematic review and meta-analysis. *Bull World Health Organ* 2014; **92**: 126–38.

17 Gillespie SH, Crook AM, McHugh TD, *et al.* Four-month moxifloxacin-based regimens for drug-sensitive tuberculosis. *N Engl J Med* 2014; **371**: 1577–87.

18 Merle CS, Fielding K, Sow OB, *et al.* A four-month gatifloxacin-containing regimen for treating tuberculosis. *N Engl J Med* 2014; **371**: 1588–98.

19 Jindani A, Harrison TS, Nunn AJ, *et al.* High-dose rifapentine with moxifloxacin for pulmonary tuberculosis. *N Engl J Med* 2014; **371**: 1599–608.
20 Menzies D, Benedetti A, Paydar A, *et al.* Standardized treatment of active tuberculosis in patients with previous treatment and/or with mono-resistance to isoniazid: a systematic review and meta-analysis. *PLoS Med* 2009; **6**.
21 Marx FM, Dunbar R, Enarson DA, *et al.* The temporal dynamics of relapse and reinfection tuberculosis after successful treatment: a retrospective cohort study. *Clin Infect Dis* 2014; **58**: 1676–83.
22 Menzies D, Benedetti A, Paydar A, *et al.* Effect of duration and intermittency of rifampin on tuberculosis treatment outcomes: a systematic review and meta-analysis. *PLoS Med* 2009; **6**: 1–18.
23 Kruk ME, Schwalbe NR, Aguiar C a. Timing of default from tuberculosis treatment: a systematic review. *Trop Med Int Heal* 2008; **13**: 703–12.
24 Hong Kong Chest Service, Tuberculosis Research Centre Madras, British Medical Research Council. A controlled trial of 2-month, 3-month, and 12-month regimens of chemotherapy for sputum-smear-negative pulmonary tuberculosis. Results at 60 months. *Am Rev Respir Dis* 1984; **130**: 23–8.
25 Badri M, Lawn SD, Wood R. Short-term risk of AIDS or death in people infected with HIV-1 before antiretroviral therapy in South Africa: a longitudinal study. *Lancet* 2006; **368**: 1254–9.
26 May M, Sterne J a C, Sabin C, *et al.* Prognosis of HIV-1-infected patients up to 5 years after initiation of HAART: collaborative analysis of prospective studies. *AIDS* 2007; **21**: 1185–97.
27 Menzies NA, Cohen T, Lin HH, Murray M, Salomon JA. Population health impact and cost-effectiveness of tuberculosis diagnosis with Xpert MTB/RIF: a dynamic simulation and economic evaluation. *PLoS Med* 2012; **9**: e1001347.
28 Wolbers M, Babiker A, Sabin C, *et al.* Pretreatment CD4 cell slope and progression to AIDS or death in HIV-infected patients initiating antiretroviral therapy - The CASCADE collaboration: A collaboration of 23 cohort studies. *PLoS Med* 2010; **7**: 1–9.
29 Rodriguez B, Sethi AK, VCheruvu VK, *et al.* Predictive Value of Plasma HIV RNA Level on Rate of CD4 T-cell decline in untreated HIV infection. *JAMA* 2006; **296**: 1498–506.
30 Lessells RJ, Mutevedzi PC, Iwuji CC, Newell M-L. Reduction in early mortality on antiretroviral therapy for adults in rural South Africa since change in CD4+ cell count eligibility criteria. *J Acquir Immune Defic Syndr* 2014; **65**: e17-24.
31 Dowdy DW, Chaisson RE. The persistence of tuberculosis in the age of DOTS: reassessing the effect of case detection. *Bull World Health Organ* 2009; **87**: 296–304.
32 Selwyn P a, Hartel D, Lewis V, *et al.* A prospective study of the risk of tuberculosis among intravenous drug users with human immunodeficiency virus infection. *N Engl J Med* 1989; **320**: 545–50.
33 Gilks CF, Godfrey-Faussett P, Batchelor BIF, *et al.* Recent transmission of tuberculosis in a cohort of HIV-1-infected female sex workers in Nairobi, Kenya. *Aids* 1997; **11**: 911–8.
34 Carvalho ACC, Deriemer K, Nunes ZB, *et al.* Transmission of Mycobacterium tuberculosis to Contacts of HIV-infected Tuberculosis Patients. *Am J Respir Crit Care Med* 2002; **164**: 2166–71.
35 Huang CC, Tchetgen ET, Becerra MC, *et al.* The effect of HIV-related immunosuppression on the risk of tuberculosis transmission to household contacts. *Clin*

Infect Dis 2014; **58**: 765–74.
36 Hermans SM, Castelnuovo B, Katabira C, *et al.* Integration of HIV and TB services results in improved TB treatment outcomes and earlier prioritized art initiation in a large urban HIV clinic in Uganda. *J Acquir Immune Defic Syndr* 2012; **60**: 29–35.
37 Ngamvithayapong J, Yanai H, Winkvist a, Diwan V. Health seeking behaviour and diagnosis for pulmonary tuberculosis in an HIV-epidemic mountainous area of Thailand. *Int J Tuberc Lung Dis* 2001; **5**: 1013–20.
38 Cohen KA, Abeel T, Manson McGuire A, *et al.* Evolution of extensively drug-resistant tuberculosis over four decades: whole genome sequencing and dating analysis of Mycobacterium tuberculosis isolates from KwaZulu-Natal. *PLoS Med* 2015; **12**: 1–22.
39 Merker M, Blin C, Mona S, *et al.* Evolutionary history and global spread of the Mycobacterium tuberculosis Beijing lineage. *Nat Genet* 2015; **47**: 242–9.
40 Lew W, Pai M, Oxlade O, Martin D, Menzies D. Initial Drug Resistance and Tuberculosis Treatment Outcomes: systematic review and meta-analysis. *Ann Intern Med* 2008; **149**: 123–34.
41 Johnston JC, Shahidi NC, Sadatsafavi M, Fitzgerald JM. Treatment outcomes of multidrug-resistant tuberculosis: a systematic review and meta-analysis. *PLoS One* 2009; **4**.
42 Holtz TH, Sternberg M, Kammerer S, *et al.* Time to sputum culture conversion in multidrug-resistant tuberculosis: predictors and relationship to treatment outcome. *Ann Intern Med* 2006; **144**: 650–9.
43 Ahuja SD, Ashkin D, Avendano M, *et al.* Multidrug resistant pulmonary tuberculosis treatment regimens and patient outcomes: an individual patient data meta-analysis of 9,153 Patients. *PLoS Med* 2012; **9**.
44 Dharmadhikari AS, Mphahlele M, Venter K, *et al.* Rapid impact of effective treatment on transmission of multidrug-resistant tuberculosis. *Int J Tuberc Lung Dis* 2014; **18**: 1019–25.
45 Toczek A, Cox H, Du Cros P, Cooke G, Ford N. Strategies for reducing treatment default in drug-resistant tuberculosis: systematic review and meta-analysis. *Int J Tuberc Lung Dis* 2013; **17**: 299–307.
46 Ahmad Khan F, Gelmanova IY, Franke MF, *et al.* Aggressive regimens reduce risk of recurrence after successful treatment of MDR-TB. *Clin Infect Dis* 2016; **63**: 214–20.
47 Borrell S, Gagneux S. Infectiousness, reproductive fitness and evolution of drug-resistant Mycobacterium tuberculosis. *Int J Tuberc Lung Dis* 2009; **13**: 1456–66.
48 Billington OJ, McHugh TD, Gillespie SH. Physiological cost of rifampin resistance induced in vitro in Mycobacterium tuberculosis. *Antimicrob Agents Chemother* 1999; **43**: 1866–9.
49 Grandjean L, Gilman RH, Martin L, *et al.* Transmission of multidrug-resistant and drug-susceptible tuberculosis within households: a prospective cohort study. *PLoS Med* 2015; **12**: 1–22.
50 Weyer K, Brand J, Lancaster J, Levin J, Walt M van Der. Determinants of multidrug-resistant tuberculosis in South Africa : results from a national survey. *South African Med J* 2007; **97**: 1120–8.
51 Nhung N V., Hoa NB, Sy DN, Hennig CM, Dean AS. The fourth national anti-tuberculosis drug resistance survey in Viet Nam. *Int J Tuberc Lung Dis* 2015; **19**: 670–5.
52 Marin J-M, Robert CP. Importance sampling methods for Bayesian discrimination

between embedded models. *Front Stat Decis Mak Bayesian Anal* 2010; : 513–27.
53 Sharma A, Hill A, Kurbatova E, *et al.* Estimating the future burden of multidrug-resistant and extensively drug-resistant tuberculosis in India, the Philippines, Russia, and South Africa: a mathematical modelling study. *Lancet Infect Dis* 2017; **17**: 707–15.
